# Application of artificial intelligence in paediatric oncology imaging

**DOI:** 10.1007/s00247-026-06606-1

**Published:** 2026-04-28

**Authors:** Giulia De Donno, Isabelle S. A. de Vries, Laura M. E. Adriaansen, Arthur J. A. T. Braat, Simone A. J. ter Horst, Geert O. Janssens, Bart de Keizer, Alexander Leemans, Johannes H. M. Merks, Rutger A. J. Nievelstein, Alida F. W. van der Steeg, Alberto De Luca, Rick R. van Rijn

**Affiliations:** 1 Division of Imaging and Oncology, University Medical Center Utrecht, Heidelberglaan 100, 3584CX Utrecht, the Netherlands; 2 Princess Máxima Center for Pediatric Oncology, Utrecht, the Netherlands; 3 Department of Radiology and Nuclear Medicine, Division of Imaging and Oncology, University Medical Center Utrecht, Utrecht, the Netherlands; 4 Department of Radiation Oncology, Division of Imaging and Oncology, University Medical Center Utrecht, Utrecht, the Netherlands; 5 Department of Radiology and Nuclear Medicine, University of Amsterdam, Amsterdam UMC, Amsterdam, the Netherlands

**Keywords:** Artificial intelligence, Children, Radiology, Oncology

## Abstract

Paediatric oncology relies heavily on medical imaging for diagnosis, treatment planning, and longitudinal disease monitoring. Yet the field faces unique challenges, including a limited number of patients, diverse anatomy, motion artefacts, and a global shortage of subspecialised radiologists. These constraints can compromise diagnostic accuracy, prolong workflows, and increase the risk of errors, highlighting a critical need for innovative solutions. Artificial intelligence (AI) has emerged as a transformative tool capable of enhancing the entire imaging pipeline. From acquisition to reporting, AI-driven methods show potential to improve image quality, correct motion artefacts, harmonise multicentre datasets, and accelerate scans while reducing radiation exposure. Deep learning models and radiomics have been shown capable of precise tumour segmentation, early lesion detection, and classification, while integration with clinical and molecular data supports individualised staging, prognosis, and therapeutic decision-making. Beyond analysis, natural language processing and large language models can streamline report generation and clinical documentation, potentially enabling more efficient communication and workflow optimisation. Despite these advances, paediatric applications remain constrained by small, heterogeneous datasets, limited paediatric-specific models, and challenges in generalisability, explainability, and regulatory approval. Strategies such as model generalisation across new datasets, the development of retrainable generic models, privacy-preserving training, and synthetic data generation can help overcome these barriers, thereby improving model robustness and promoting equity in care. By augmenting rather than replacing radiologists, AI holds the potential to transform paediatric oncology imaging, improving diagnostic precision, workflow efficiency, and enhancing access to high-quality care. Continued collaboration between clinicians, data scientists, and regulatory bodies will be essential to realise this promise safely and effectively.

**Graphical abstract:**

**Figure:**
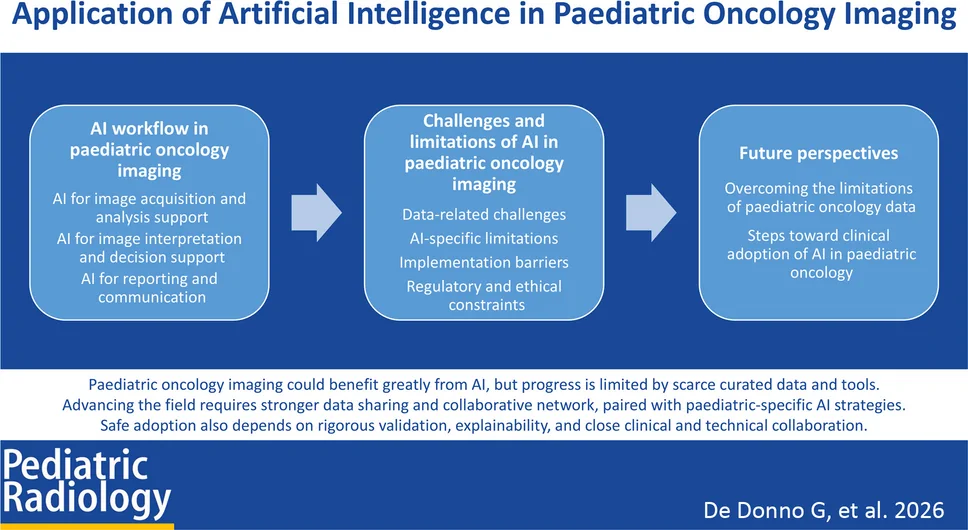

**Supplementary Information:**

The online version contains supplementary material available at https://doi.org/10.1007/s00247-026-06606-1.

## Introduction

According to the World Health Organization (WHO), each year approximately 400,000 children and adolescents between 0 years and 19 years develop cancer worldwide, making it by definition a rare disease [1, 2]. As a result, most clinicians, paediatricians, and radiologists will encounter only a small number of paediatric cancer patients in their careers unless they work in a specialised paediatric oncology centre. Furthermore, imaging in the oncology setting requires extensive knowledge of the underlying tumour biology, characteristic imaging features, and the treatment approach for optimal interpretation. These challenges are particularly relevant when dealing with rare diseases such as those in paediatric oncology, where the small number of patients makes standardised imaging assessment more difficult. Consequently, an intensive collaboration between paediatric radiologists, oncologists, surgeons, radiation oncologists, and pathologists is essential to achieve an optimal treatment outcome [3]. The situation is further complicated by a global shortage of radiology subspecialists, even as imaging demands continue to rise [4–8]. This shortage is particularly problematic in paediatric oncology, where the rarity of the disease and the diverse international imaging and reporting guidelines create additional demands for specialised expertise [9, 10].

By enabling large-scale multicentre analyses, the era of big data and artificial intelligence (AI, Table 1) offers a promising way to overcome these limitations [6, 11]. While radiologists are able to recognise many disease patterns, AI can aid in detecting subtle or rare variations, uncovering clinically relevant effects that might be missed by individual observers, as well as reducing inter- and intra-observer variability. Furthermore, AI excels in handling repetitive or complex tasks and combining multiple types of data with consistent accuracy. This ability to quickly process vast amounts of information consistently can relieve humans from tedious or error-prone tasks [10, 12–14].

Given the complexities of paediatric oncology imaging, AI holds significant potential to support clinicians and improve quality of care across the entire workflow. This review examines the current research landscape and some future directions. It highlights the gap between the large body of research and its limited real-world adoption, as well as intrinsic limitations of paediatric data and AI, alongside strategies to address them. Ultimately, the aim is to enable the safe and effective use of AI in areas where it can have the greatest impact.

## Artificial intelligence workflow in paediatric oncology imaging

This overview examines the potential transformative role of AI in paediatric oncology imaging, from image acquisition and preprocessing to interpretation, clinical decision support, and reporting. Throughout the diagnostic process, AI can support clinical tasks while aiding error detection and optimising workflow, ultimately enhancing accuracy and ensuring high-quality care for paediatric oncology patients [9, 12, 17]. While the review focuses on paediatric studies, selected findings from adult or general population research are also included when they provide relevant insights translatable to paediatric oncology. A table classifying the sources by paediatric and adult populations is provided in Supplementary Material 1, and the full methodology in Supplementary Material 2. AI terms used in this manuscript are briefly explained in Table 1.

**Table 1:** Overview of artificial intelligence-related terms described in this publication, with examples of use in the context of image segmentation

Artificial intelligence (AI)	Overarching term that includes machine learning and deep learning approaches. It refers to a set of algorithms capable of tasks like pattern recognition and decision-making. Unlike traditional programming, where a developer explicitly defines every rule, AI systems may learn the underlying patterns and relationships directly from data, enabling them to process complex inputs (such as a medical image) and generate a corresponding output or decision Example: A program examines a medical image and identifies potential tumours either by applying established rule-based criteria (e.g. intensity, shape, texture thresholds) or by using data-driven features
Radiomics	Radiomics refers to the extraction of measurable features such as texture, shape, and intensity from the image. These features can capture information not visible to the human eye and provide a structured representation of the image that can be used as input for machine learning models. In conventional radiomics, features are usually manually defined based on expert knowledge Example: Shape, volume, and texture features are extracted from a segmented tumour and used as input for a machine learning model
Machine learning (ML)	A subset of AI in which algorithms learn patterns from labelled examples to make predictions or classifications. The system requires training and improves as the dataset becomes larger or more representative. In medical imaging, ML often uses numerical data derived from images (e.g. radiomics features or other measurements) rather than analysing the raw images directly. Clinical data can also be incorporated Example: A *k*-nearest neighbours algorithm is trained on radiomics features extracted from previously labelled tumours and normal tissue, and then used to classify new voxels in a scan based on their similarity to the training examples
Deep learning (DL)	A subset of ML that automatically learns relevant features directly from raw data without the need for manual selection of features. It is called “deep” because these networks consist of multiple layers that capture increasingly abstract representations Example: A network trained on annotated scans automatically segments tumours end-to-end, learning features directly from the images
Convolutional neural network (CNN)	A type of DL model particularly suited for image analysis. CNNs use convolutional layers to automatically learn important patterns from image data, such as edges, textures, and shapes, which helps them identify and segment regions of interest without the need for manually defined features Example: After training, a nnU-Net [15] model is used to automatically segment tumours in the image
Generative adversarial network (GAN)	A DL architecture composed of two neural networks (in the context of medical imaging, usually CNNs): a generator that creates synthetic images and a discriminator that evaluates their realism. The 2 networks compete and improve each other over time. They can be used for image enhancement, improving low-resolution or noisy images, image-to-image translation between modalities, and generation of realistic synthetic images to expand limited datasets while preserving patient privacy Example: When a specific class of images (e.g. a certain age group or tumour subtype) is underrepresented, a GAN is used to generate additional samples for that class
Transfer learning	A learning strategy in which a model pre-trained on one task or dataset is adapted to a related task, often using fewer labelled examples. This approach is particularly useful when labelled data are scarce Example: A neural network pre-trained on adult imaging data is fine-tuned to segment paediatric tumours, improving performance when training data are limited
Supervised, weakly supervised, unsupervised, or self-supervised learning	Different training approaches use different amount of labelled data. Most models are trained using supervised learning, where each image is paired with a reference label. This approach typically achieves high task-specific accuracy but requires substantial manual effort. Recent methods have shown that accurate segmentations can also be learned from incomplete, inexact, or noisy annotations (weakly supervised learning) or even entirely unlabelled datasets (unsupervised or self-supervised learning). These methods reduce the need for manual annotations, though they may require more complex models and can sometimes achieve slightly lower task-specific accuracy Example: A self-supervised model learns anatomical representations from unlabelled scans and is later fine-tuned using a small labelled paediatric dataset
Foundation model (FM)	Extremely large pre-trained models that can be adapted (typically via fine-tuning) for a wide range of downstream tasks such as segmentation or classification. These models are trained on massive (sometimes multimodal) datasets, making training from scratch computationally demanding Example: A foundation model such as TotalSegmentator [16] is used to perform a generic task (e.g. segmentation of healthy kidneys) without task-specific training from scratch
Large language model (LLM)	An AI model trained on massive amounts of text to understand and generate human-like language. LLMs can answer questions, summarise text, translate languages, or generate structured reports by predicting the most likely words or sentences based on context. In medicine, they can help interpret clinical notes, draft radiology reports, or assist in decision support Example: Following image segmentation or tumour classification, an LLM is used to generate a structured radiology report containing relevant findings

*AI* artificial intelligence, *CNN* convolutional neural network, *DL* deep learning,* FM* foundation model,* GAN* generative adversarial network,* LLM* large language model,* ML* machine learning

### Artificial intelligence for image acquisition and analysis support

#### Scan time reduction

Infants and children often require general anaesthesia to reduce motion and ensure comfort during scans, although this introduces risks and additional costs. Older children may still move or experience discomfort, though usually to a lesser extent [12]. Minimising scan time while maintaining high-quality images with good contrast, spatial resolution, and signal-to-noise ratio is essential for accurate diagnosis and treatment. Consequently, as paediatric oncology patients often require frequent scanning, minimising radiation exposure in computed tomography (CT) and X-rays and addressing reconstruction limitations remain key challenges [12, 18]. There are a few ways in which AI methods have shown promise to improve paediatric image acquisition while reducing scan time.

A first approach involves the personalisation of image acquisition, where AI algorithms could help adapt CT protocols to individual patient characteristics such as age, body mass index, medical history, and clinical indication. Such methods could support the automated selection of scan parameters, positioning, and range to optimise image quality while limiting unnecessary exposure [18, 19]. By incorporating prior information and patient-specific features, these approaches may assist in minimising cumulative radiation doses for children who undergo multiple scans while reducing operator variability in routine clinical workflows.

A second approach focuses on image acceleration, a long-standing field of research in both magnetic resonance imaging (MRI) and CT aimed at reducing motion artefacts, CT radiation dose, and sedation requirements. The basic principle involves acquiring data more rapidly, often at lower quality or with fewer samples, and subsequently restoring image fidelity during or after reconstruction. In MRI, compressed sensing has traditionally been used to reconstruct high-quality images from under-sampled data, while in CT, iterative reconstruction algorithms can produce diagnostically useful images from low-dose acquisitions. More recently, deep learning–based reconstruction has shown potential, including in paediatric applications, to achieve similar or improved image quality at lower doses and faster acquisition times [12, 18, 20, 21]. For example, initial studies on paediatric chest and abdomen CT have reported dose reductions of 36–70% without losing diagnostic information [22] and show great potential in low-dose head, temporal bone, lung, spine, and abdominal CT, as well as CT angiography [23].

Complementary to these reconstruction approaches, AI-based post-processing and denoising methods have also shown potential to improve image quality from low-dose or accelerated acquisitions. These techniques operate on reconstructed images to suppress noise, reduce artefacts, and enhance structural detail, thereby improving downstream analyses. Denoising is particularly relevant in paediatric imaging, where lower radiation doses often lead to noisier images [18]. Deep learning models (DL, Table 1), most notably convolutional neural networks (CNNs, Table 1) and generative adversarial networks (GANs, Table 1), have demonstrated the ability to substantially reduce image noise and restore visual quality comparable to full-dose images [12, 18, 24]. These methods may also help reduce reliance on contrast agents and further accelerate image acquisition. For instance, DL approaches have achieved a 90% to 100% reduction in gadolinium-based contrast agent dose for adult patients with a wide range of clinical indications and brain abnormalities. These models synthesise full-dose brain MR images from pre-contrast and ultra-low-dose inputs, reducing the potential adverse effects of the contrast agent [25, 26]. CNNs and GANs have also been explored to generate paediatric total-body high-dose positron emission tomography (PET) images from low-dose data, for example by combining short-duration PET acquisition with low-dose CT information, potentially reducing tracer dose and scan time [27]. Similar approaches have been applied to ultra-fast low-resolution MRI, enhancing resolution and reducing noise, though most methods are adapted from adult datasets and require further validation in paediatric populations [12, 28]. Other paediatric applications in MRI include DL-based super-resolution, which can reconstruct high-resolution three-dimensional (3D) images from fast, low-resolution two-dimensional (2D) brain scans, outperforming simple interpolation in visual quality [29].

Beyond denoising, AI-supported post-processing can further enhance image quality through motion correction, artefact reduction, automated quality assessment, and contrast enhancement techniques. Motion correction approaches may be applied prospectively during image acquisition or retrospectively during post-processing to estimate and compensate for patient movement [30]. Although most AI-based motion correction strategies have been validated primarily in adults, applications in foetal MRI suggest that DL models can identify anatomical landmarks, estimate motion, and reconstruct more consistent images [31, 32]. CNN- and GAN-based models have been proposed also for children to detect and correct artefacts caused by metal, motion, or low-dose imaging in X-ray, CT, and MRI, often achieving better visual outcomes than traditional approaches [12, 18, 28]. Automated quality assessment models may further assist by identifying low-quality slices for reacquisition, thereby improving dataset reliability [31]. AI-driven contrast enhancement techniques, such as histogram equalisation and adaptive contrast enhancement, can help improve tissue differentiation and diagnostic visibility, improving classification accuracy and segmentation performances [11, 12, 18].

Finally, another way to decrease scan time is predicting additional sequences or modalities from already acquired data through synthetic image generation. Image-to-image translation methods are widely investigated, especially in the adult population, with nearly half of the studies focusing on generating other modalities from MRI, a quarter on Cone-Beam CT-to-CT, and smaller proportions on PET attenuation correction or low-dose to full-dose CT conversion [33]. MRI-to-CT synthesis could be particularly valuable in interventional radiology and radiotherapy planning, where synthetic CT derived from MR data may reduce the need for separate CT acquisitions. Preliminary studies in paediatric abdominal imaging have shown that synthetic CT can be acceptable for photon and proton therapy planning [34], though further validation is required before diagnostic adoption. These techniques may also be beneficial in paediatric patients who are sensitive to radiation, including those with genetically radiosensitive cancer predisposition syndromes, and those who may need repeated imaging [33, 35, 36]. Similarly, studies in adults have shown that synthetic CTs could be generated directly from the PET scan to serve as an attenuation map, eliminating the need for a separate CT. This approach not only reduces overall radiation exposure for the patient but also mitigates common CT-related artefacts, such as those caused by patient motion or metallic implants [37]. Other applications include generating synthetic CT from orthogonal X-rays to improve the automatic prediction of paediatric abdominal anomalies while reducing radiation exposure [38]. Beyond cross-modality translation, MRI-to-MRI synthesis is used to generate MR sequences or contrasts directly from existing acquisitions. Convolutional and generative models, such as GAN-based frameworks (e.g. CycleGAN, Pix2Pix), have been used to synthesise standard clinical sequences like fluid-attenuated inversion recovery (FLAIR), T1-weighted (including contrast-enhanced), and T2-weighted sequences from one another, also in paediatric populations [39–42]. Compared with traditional physics-based methods, AI-based methods have demonstrated higher overall image quality in MRI-to-MRI synthesis in paediatric brain imaging, while maintaining equivalent scan times [39]. Beyond clinical use, synthetic image generation can also support data augmentation for AI training, helping mitigate the limited availability of paediatric imaging data and address privacy constraints [33, 36].

#### Image harmonisation

As childhood cancer is a rare disease, studies require international multicentre research. This introduces variability into data due to differences in imaging protocols and scanners [43] potentially causing biased AI models and reducing generalisation. Harmonisation addresses the issue by standardising image appearance and characteristics through methods such as grayscale normalisation, resampling, colour normalisation, denoising, and contrast enhancement. This enables fair comparisons and improves the performance of an AI model. While statistical approaches have traditionally dominated, the application of DL methods has grown since 2017 due to their ability to handle complex variability, although their development remains largely confined to adult patient studies. To improve robustness, hybrid models combining both approaches have been introduced [11]. In PET, harmonisation has been facilitated by standardised accreditation programmes, such as the European Association of Nuclear Medicine (EANM) Research Ltd. (EARL) certification [44], which enforces consistent reconstruction and quantification protocols across centres. This framework enables reproducible multicentre PET studies and provides a more stable foundation for AI model development and validation. For retrospective data where these requirements were not originally met, AI models can now be used to detect non-compliant images and support harmonisation [165]. Similar accreditation schemes are currently lacking for MRI, although several initiatives have proposed guidelines and strategies to uniform acquisitions in specific fields and reduce variability of quantitative methods such as the apparent diffusion coefficient (ADC) from diffusion MRI. In the future, harmonisation on both the acquisition (by standardising acquisition protocols) and post-processing side (using AI) promises to facilitate the clinical comparability of MRI acquisitions across sites [45, 46].

#### Registration

Image registration aligns images across time, modalities, and individuals for applications such as clinical diagnosis, radiation therapy planning, and morphometric analysis [47]. In oncology, registration enables the integration of multimodal information from MRI, PET, and single-photon emission computed tomography (SPECT) to correlate dose distributions with imaging biomarkers, assess treatment effects, and evaluate tumour metabolism [48, 49]. Accurate registration is also essential for creating population-based templates and atlases, providing a spatial framework for multicentre and longitudinal studies [12]. Its role is also expanding in paediatric surgical oncology, where high-resolution CT and MRI are integrated with 3D reconstruction, cinematic rendering, and virtual or augmented reality to support complex surgical planning. These innovations provide new insights into paediatric tumour anatomy and treatment response, contributing to more individualised and precise therapeutic strategies [50].

Conventional registration methods typically optimise deformation fields in a coarse-to-fine manner, leveraging global and local information. Despite being a well-established tool in medical image processing, accurate registrations remain challenging to achieve, particularly between images with different fields of view or contrasts. DL approaches, often using CNNs, have recently demonstrated superior robustness and computational efficiency, offering faster and more consistent multimodal alignment [10, 47]. Patch-wise strategies further enhance performance by capturing localised deformations while reducing memory demands, improving registration accuracy even when training data are limited [47]. DL-based synthetic imaging can help overcome inter-modality differences in registration. For example, direct registration between CT and MR images can be challenging due to their different contrast characteristics. By generating a synthetic CT from MRI, the two images become more similar in appearance, effectively converting an inter-modality registration problem into an intra-modality one and improving overall alignment accuracy [33, 51].

Despite these advances, all the reported examples of AI-based registration methods were evaluated in adults, whereas registration in paediatric imaging remains severely understudied. Differently from adults, registration in the paediatric domain will also have to consider the unique challenges of this population due to physiological growth, disease- and surgery-related deformation, and heterogeneous imaging quality, particularly in longitudinal studies where images are acquired over several years. Altogether, this highlights the need for methods capable of adapting to anatomical and temporal variability [52].

### Artificial intelligence for image interpretation and decision support

AI is transforming paediatric oncology imaging by uncovering hidden patterns, detecting subtle abnormalities and anticipating individual treatment responses. By integrating detection, segmentation, classification, and longitudinal monitoring, it can enhance diagnostic precision, support clinical decisions, and enable more personalised care for children.

#### Detection of abnormalities

In imaging, anomalies represent deviations from normal structural or functional patterns and may signal a wide range of conditions, from tumours to organ dysfunction [24]. For instance, surveillance screening has been used in children to detect subtle brain alterations in neurodevelopmental disorders, as well as cancer predisposition syndromes which can improve early tumour detection and, consequently, survival rates [12]. Traditionally, detecting abnormalities in medical images relies on radiologists’ expertise. Early computer-aided detection attempted to formalise this with predefined mathematical rules, but it was often task-specific and limited in accuracy [10]. Radiomics (Table 1) extends this approach by extracting predefined quantitative imaging features reflecting tumour heterogeneity and spatial patterns but requires careful preprocessing.

In contrast, AI methods can automate feature selection, making abnormality detection more robust and reproducible [24]. DL models like CNNs, GANs, and hybrid approaches analyse CT, MRI, and X-ray images more consistently, detecting subtle abnormalities that support earlier diagnosis and treatment planning [17, 24]. These networks have been used for classification, subtype distinction, and improved detection of lesions in adult brain and lung tumours [53, 54]. AI can also integrate imaging with clinical and genomic data to predict treatment response and guide personalised therapy to maximise efficiency and minimise side effects [12, 17].

#### Segmentation

**Fig. 1:**
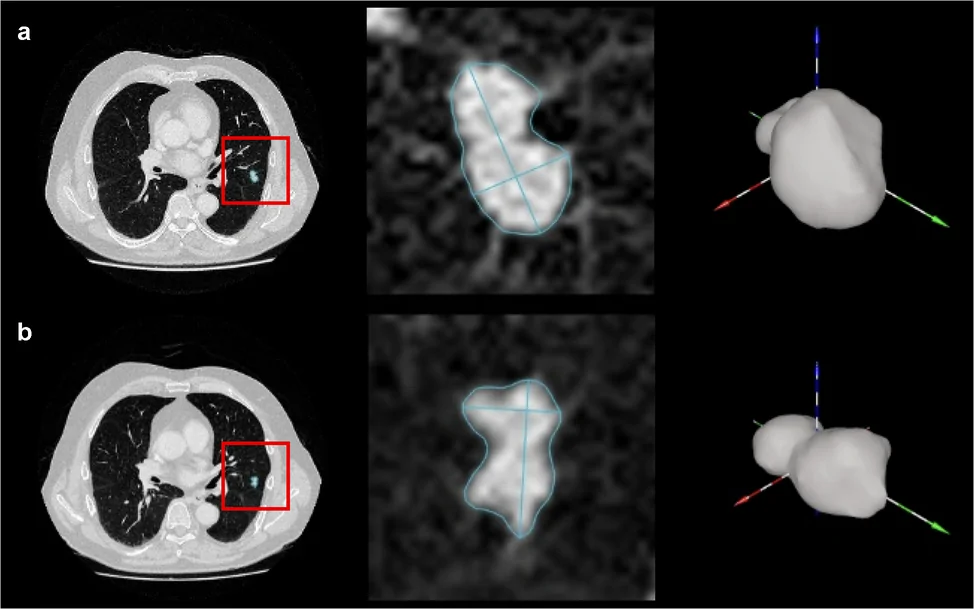
Axial non-contrast chest CT of an adult male patient, with AI-based pulmonary nodule detection (Veye Lung Nodules, version 3.26.3; DeepHealth, Amsterdam, NL) at diagnosis (**a**) and after 4 months (**b**). At diagnosis, AI annotation (*inside the red square*) highlights a solitary nodule in the left upper lobe, demonstrating an approximate 44% reduction in volume at response evaluation (courtesy of A.J. Hoving, MD, PhD, Department of Radiology, Spaarne Gasthuis, Haarlem, the Netherlands). *AI* artificial intelligence, *CT* computed tomography

**Fig. 2:**
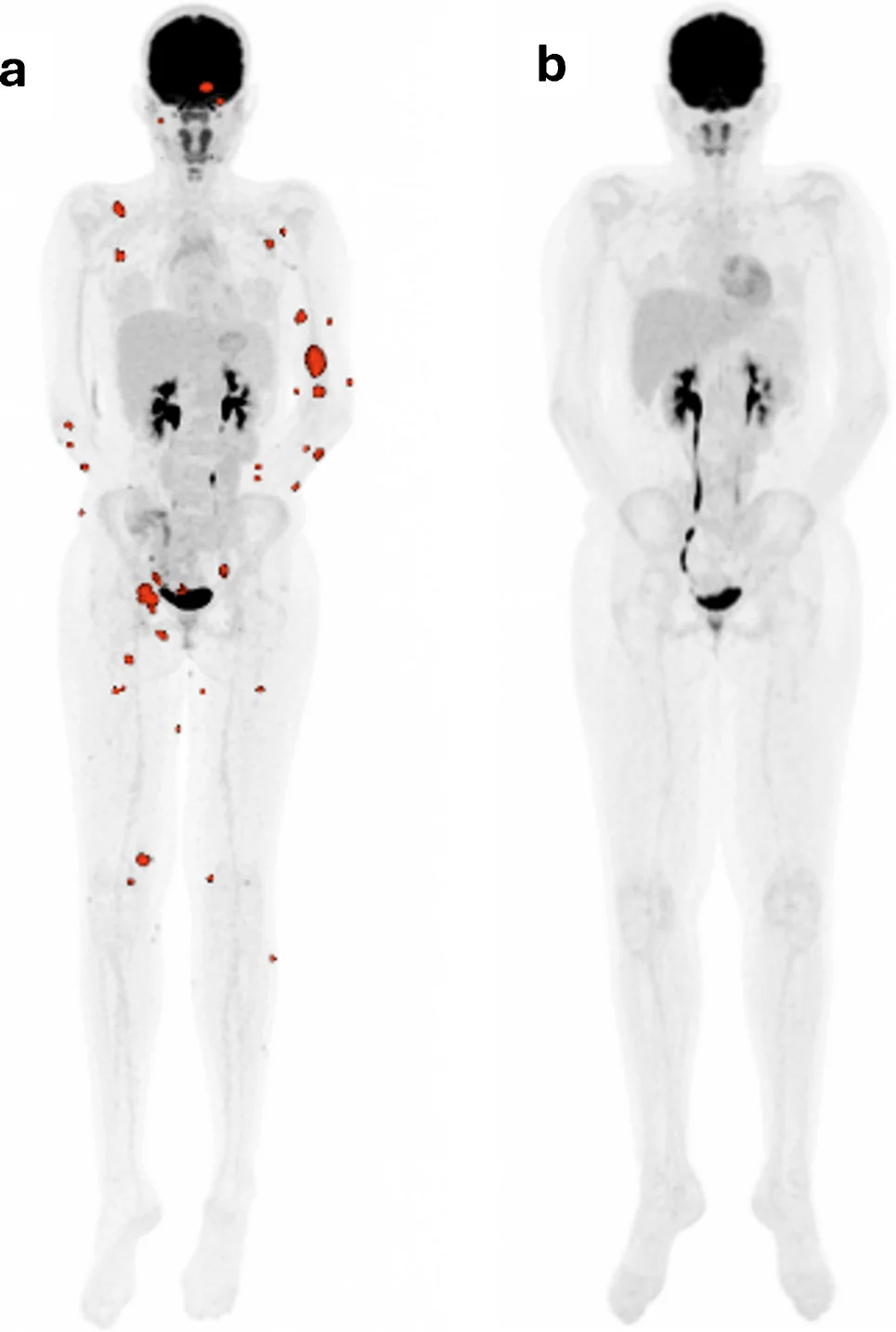
A fourteen-year-old girl with anaplastic large cell lymphoma, a subtype of non-Hodgkin lymphoma, with cutaneous and lymph node localisations. [18F]FDG-PET/CT was acquired at initial diagnosis (**a**) and after three cycles of chemotherapy (**b**). The interim response demonstrates a complete metabolic response, i.e. with all lesions’ metabolic activity becoming less than or equal to blood pool activity (Deauville score 2). Automated nnU-Net-based segmentation tool easily segmented all disease localisations on the baseline scan (**a**) and did not find any comparable lesions on interim response imaging (**b**). As a result, all baseline lesions were considered to be in complete remission. In line with the developments in adults, the field gradually tends toward total metabolic tumour volume quantification, as demonstrated by Boellaard et al. [69]. Artificial intelligence is expected to improve clinical implementation of these new imaging biomarkers. *[18F]FDG-PET/CT* fluorine-18 fluorodeoxyglucose positron emission tomography/computed tomography

Medical image segmentation classifies each voxel in an image into binary or multiclass regions and is an important step for many aspects of image interpretation and decision support. Segmentation in oncology enables accurate delineation of tumours for a variety of clinical purposes. Measurements of tumour size and volume allow assessment of disease progression and therapy response, supporting informed decisions regarding next steps in treatment (e.g. Fig. 1, Fig. 2). In radiotherapy, segmentation guides dose delivery to maximise tumour irradiation while avoiding organs and structures at risk [55]. Automated segmentation methods have been widely applied across paediatric care, for example in the spine and other musculoskeletal structures and thoracic, abdominal, and pelvic organs, as well as numerous neuroimaging applications. These models address diverse clinical needs, both in healthy and abnormal anatomy caused by injuries, tumours, and other anomalies [12, 14, 28]. In surgical planning and navigation, for example, creating 3D models of a tumour and its relationship to critical blood vessels and nerves helps surgeons determine the optimal approach to achieve complete tumour resection while preserving healthy tissue, which can improve outcomes and reduce complications [56, 57]. These models were used to successfully guide minimally invasive robotic surgery for neuroblastic, kidney, endocrine, and other types of paediatric tumours in over a hundred procedures, aiding in precise dissection and healthy tissue preservation [58]. Segmented regions also enable radiomics analyses, providing quantitative features that feed predictive models for prognosis, therapy response, and risk stratification. Moreover, standardised segmentation provides reproducible metrics across studies, facilitating multicentre analyses and evaluation of novel therapies [10, 12, 53, 59–61].

Traditional methods rely on handcrafted features such as thresholding, edge detection, and region growing, which are sensitive to noise and imaging variability and generalise poorly [62, 63]. In contrast, DL methods can leverage rich image features to achieve high-accuracy segmentation. This can provide robustness even for tumours with poorly defined margins, where distinguishing tumour tissue, oedema, non-enhancing regions, and cystic components is challenging, especially during surgical navigation or radiotherapy [12, 24, 59, 64].

Segmentation can be performed manually, semi-automatically, or fully automatically, depending on the level of human input. Manual segmentation, performed entirely by human experts, is considered the reference standard for accuracy, although it is notoriously time-consuming and suffers from inter-observer variability. Semi-automatic segmentation combines automated methods with user guidance, where input such as scribbles, bounding boxes, or clicks is used to initialise or refine the segmentation process [60, 62]. Fully automatic segmentation is the most commonly used approach, as it produces segmentations directly from imaging data without any need for human intervention.

All of these approaches can be trained using supervised, weakly supervised, or unsupervised/self-supervised learning (Table 1), depending on how much labelled data are available and the desired trade-off between annotation effort and model complexity [60, 63, 65].

Most segmentation approaches are CNN-based. For example, U-Net [66] and the self-configuring nnU-Net [15] are fully automatic models typically trained in a supervised learning setting, and they have been successfully applied to many image segmentation tasks. More recently, large foundation models (FMs, Table 1) such as MedSAM [67] and TotalSegmentator [16], though originally trained on adult populations, have been adapted via transfer learning (Table 1) to perform segmentation tasks on paediatric data [68].

#### Diagnosis and classification

Diagnosis and classification of tumours using AI have the potential to become integral in paediatric oncology care, complementing radiologists’ assessments and enabling early, accurate identification of complex conditions [9, 10]. Early and precise tumour characterisation is critical for improving outcomes, yet interpreting complex imaging data in children remains challenging due to developmental variability and overlapping radiological features. Machine learning (ML, Table 1) models can potentially differentiate benign from malignant lesions by integrating clinical, demographic, imaging, and genomic data [53, 60].

A key step in tumour assessment is the analysis of imaging features that reflect the underlying biology of lesions. Quantifying features such as size, texture, and enhancement patterns can reveal clinically relevant information about tumour type. Radiomics extracts such features, often called imaging biomarkers, from MRI, CT, and PET scans to support diagnosis, monitoring, and treatment planning [17, 70]. In paediatric Ewing sarcoma, for instance, MRI-based radiomics can predict chemotherapy response and tumour subtype, while PET radiomics adds metabolic and microenvironmental data that enhance assessment accuracy in assessing tumour behaviour [17].

DL further expands feature analysis by automatically learning complex patterns from imaging data. CNNs have shown strong performance in tumour classification and segmentation, often exceeding human accuracy while reducing diagnostic time and errors. A review of 102 studies found that most CNN-based models, especially hybrid or ensemble designs, achieved over 90% accuracy for adult brain tumour classification [53]. In a multicentre paediatric study, a DL model distinguished medulloblastoma from pilocytic astrocytoma with 97% accuracy, performing on par with expert neuroradiologists while surpassing less experienced readers [71]. Strong results have been reported in other paediatric applications, with AI models achieving high performance in osteosarcoma, Wilms tumour, pulmonary nodules, lymphoma, soft-tissue sarcomas, and multiple types of brain tumours [28].

#### Staging and prognosis

Staging systems, such as the tumour-node-metastasis (TNM), classify patients into predefined categories based on measurements from imaging, providing a standardised assessment of tumour extent. Response Evaluation Criteria in Solid Tumors (RECIST) [72] and WHO [73] criteria simplify evaluation by reducing the cognitive burden for clinicians [10]. Stratification criteria, however, differ by tumour type and include factors such as location, histology, size, and molecular profile. This variability makes accurate assessment especially challenging for rare tumours and highlights the need for AI-assisted tools [54]. Staging is fundamental to guiding treatment decisions and determining prognosis, which reflects the expected course of disease, including survival and likelihood of recurrence.

ML and DL models can further integrate clinical, genomic, and imaging data to stratify patients, inform treatment intensification or de-escalation, and predict overall survival and recurrence [17]. In a review of fifty studies on survival prediction for brain tumours, MRI-based AI models were reported to demonstrate strong performance in classifying survival groups (short-, mid-, and long-term) and estimating survival times [74]. In this review, most high-performing models focused on glioblastoma and glioma in adult populations, with paediatric applications being rare. A notable exception was a paediatric study (mostly focused on medulloblastoma and pilocytic astrocytoma) [75] which demonstrated promising results by achieving exceptional accuracy for survival group classification [74].

Furthermore, these models provide prognostic insight into diagnosis, particularly for unresectable tumours, supporting risk-adapted interventions [12]. By predicting treatment response and optimising therapeutic regimens, AI and radiomics show direct promise for informing personalised treatment planning [9]. This paradigm of using predictive models to tailor therapies is crucial: evidence shows that patients who receive more accurate prognoses and subsequent personalised treatments experience significantly better outcomes. For instance, early diagnosis of lung cancer with appropriate treatment improved survival in adults by 20% [9, 54, 76].

#### Monitoring treatment response

Imaging is a key asset to monitor treatment response. For neuro-oncology, the Response Assessment In Pediatric Neuro-Oncology (RAPNO) group has published two in-depth reviews on this topic [77, 78] highlighting both the potential of AI to enhance quantitative, reliable assessment of treatment response and the significant challenges that still hinder its clinical adoption.

In glioma patients, for example, recurrence is difficult to predict with conventional clinical or genomic markers. Longitudinal DL analysis of paediatric MRI scans has shown substantial improvements in individualised recurrence prediction for both low- and high-grade gliomas, with reported performance gains of up to 58% compared with traditional approaches [79]. Furthermore, DL models combining imaging and clinical features in paediatric low-grade gliomas have been shown to improve postoperative risk stratification and event-free survival prediction, supporting more precise monitoring and management strategies [80].

To accurately assess tumour response, it is important to differentiate between true tumour progression and treatment-related inflammation, a task where ML models have shown promising success [12]. Furthermore, initial research explores the prediction of complications, such as those related to chemotherapy, which could help optimise patient management plans [76, 81]. While these results are promising, they highlight a growing area of research rather than established clinical practice.

### Artificial intelligence for reporting and communication

Radiology reports are critical in oncology, but they are time-consuming to write and can contain errors. Furthermore, incomplete clinical histories and unstandardised communication between radiologists and oncologists can lead to missed findings, inconsistent interpretations, and delayed diagnoses [10, 82]. Although electronic health records contain vast amounts of valuable information, their complexity often makes them impractical for real-time clinical use, contributing to cognitive overload and inefficiency [82].

Large language models (LLMs, Table 1) offer a promising solution to these challenges. LLMs can extract and summarise relevant clinical context from electronic health records, generate structured and narrative reports, classify unstructured text and standardise terminology, ultimately improving clarity, completeness, and workflow efficiency [13, 14, 83]. Recent studies have shown that radiologists overwhelmingly preferred LLM-generated clinical histories over standard requisitions [82], and incorporating LLMs such as LiverAI reduced radiologists’ workload by 45% while maintaining high diagnostic accuracy [13, 84].

In large multicentre studies, data is mainly extracted from case report forms that are usually completed manually by data managers. Because different studies often require distinct measurements or apply different response criteria (such as volumetric response compared with RECIST 1.1), inconsistency in data collection is common. AI support in generating tumour- or study-specific structured reports could promote greater uniformity and reproducibility of study data across institutions [85].

Beyond research applications, multimodal LLMs can integrate imaging and clinical data to generate radiology reports directly from images and vocal recordings, and to produce patient-friendly summaries that support understanding and engagement in treatment decisions [8, 13, 83, 86, 87]. These systems can also identify transcription and laterality errors, flag imaging artefacts, and suggest protocol adjustments, which is particularly valuable in rare paediatric oncology settings where radiologists encounter more specialised imaging protocols infrequently. In addition, LLMs can optimise workflow across scheduling, triage, interpretation, and follow-up, thereby improving radiologist efficiency and overall quality of patient care [13, 14, 83].

## Challenges and limitations of artificial intelligence in paediatric oncology imaging

### Data-related challenges in paediatric oncology artificial intelligence

AI development in paediatric oncology imaging faces the well-known constraints of paediatric imaging in general, including small patient populations, considerable anatomical and physiological variability, ethical restrictions, and low economic incentives [6, 9, 12, 14, 88]. These limitations are compounded by the scarcity of open-access datasets: although children make up about 30% of the world’s population, they account for only ~5% of imaging studies in the USA and globally constitute less than 1% of subjects in publicly available datasets [36, 89]. Moreover, imaging data are often not curated or are incomplete, as only a few studies have systematically collected full imaging datasets, while most rely on case report forms that record only minimal summary information rather than complete scans. Despite several ongoing research initiatives, there remains a notable scarcity of imaging data that are findable, accessible, interoperable, and reusable (FAIR), as many study networks still limit access to primary research groups. This lack of accessible and harmonised data restricts the development and validation of robust paediatric AI models, further widening the gap between technical innovation and clinical implementation [9, 89].

The scarcity of AI tools validated for paediatric use creates a clinical dilemma, leading practitioners to consider adult-trained models as a potential solution. This is a common scenario in fields like neuroimaging, where most available algorithms are designed for adult conditions. However, this approach carries significant risk, as these models frequently fail to generalise to paediatric patients. Performance degradation occurs due to fundamental differences in disease presentation and use of different imaging strategies and protocols between adults and children, as well as the developmental physiology of younger patients [14, 68, 89, 90]. Therefore, we argue that a strategic approach is needed: while adult data and frameworks can offer a foundational starting point, the focus must be on proactively addressing paediatric-specific challenges like data scarcity and developmental variability [14, 68, 91].

### Artificial intelligence-specific limitations in paediatric oncology imaging

In paediatrics, developmental variation and rare conditions create highly specialised subpopulations, often requiring dedicated models for specific diseases or age groups [9, 92]. Models must be trained on the same type of population they will be applied to, or their predictions may be unreliable or unsafe [93]. When models are not exposed to sufficient data diversity, they can become sensitive to technical variations. Differences in acquisition parameters, scanners, and institutional protocols can produce variability in brightness and contrast, distorting image features even within the same anatomy [13]. Such variability can introduce bias and potential misclassification, undermining generalisability. The consequent risk that algorithms may fail on unseen cases after performing well in controlled settings raises significant concerns about bias and ethical implications in clinical deployment [9, 12, 13, 83, 93].

Addressing these limitations requires careful attention to data quality. DL methods tend to be sensitive to imperfect labels and a lack of standardisation (especially in small models), making rigorous data curation such as consistent formats, resolution normalisation, artefact removal, and accurate labelling essential. This is especially critical in multicentre or multi-scanner studies, where curated data improves consistency in performance metrics, increases statistical power, and enhances the model’s generalisability [13, 14].

Even with carefully curated data, AI models can still produce confident yet incorrect or misleading outputs, a phenomenon also known as “AI hallucinations”. This flaw presents a critical safety concern, as users may develop automation bias, an uncritical trust that causes them to defer to the AI’s suggestion while dismissing their own expertise or contradictory data. This tendency to anthropomorphise AI or treat it as infallible can directly compromise clinical judgment (especially in less experienced users) and patient safety. Safeguarding against these dangers requires rigorous validation of AI tools, their standardisation into clinical protocols, and comprehensive training to ensure healthcare professionals remain engaged critical decision-makers [13, 83].

### Implementation barriers

Despite initial enthusiasm, a significant “AI chasm” exists between the development of models in research settings and their widespread adoption in clinical radiology practice [6], a gap illustrated by a systematic review on AI operationalisation in adult intensive care units, which found that only 2% (*n*=25) of studies progressed to clinical testing and none to full implementation [94]. At the beginning of 2026, only 5 of 281 (1.8%) CE-marked AI-based radiology products available in Europe were developed specifically for paediatric populations, and none addressed paediatric oncological applications [95]. Similar trends are observed in the USA, where data from the American College of Radiology indicate that only 32 of 448 (7%) AI products approved by the Food and Drug Administration (FDA) are tailored to paediatric use [96]. Together, these findings highlight a critical lack of clinically implemented AI solutions for paediatric radiology, despite extensive research activity in the field.

Several barriers hinder the implementation of AI paediatric radiology. Firstly, although large volumes of medical data exist, they are rarely curated and are predominantly derived from adult oncological populations, creating a major bottleneck for developing reliable paediatric AI models. Reliable curation requires trained personnel and is both time-consuming and costly [10, 13]. Integrating AI tools into existing electronic health records and clinical workflows also poses technical and logistical challenges, especially in resource-limited settings [9]. Furthermore, DL models demand substantial computational resources, such as high-performance graphics and tensor processing units or cloud-based infrastructure, which can be expensive to acquire and maintain. Even in well-resourced centres, continuous software updates, model revalidation, and integration into clinical workflows require ongoing investment. Moreover, as software packages evolve, trained models risk becoming obsolete unless actively maintained, raising critical questions about responsibility and long-term sustainability in clinical environments [18]. Similarly, although data sharing offers a way to accelerate research and improve model generalisability, it requires robust governance frameworks, secure infrastructure, and clear lifecycle management strategies. Developing and maintaining data commons is expensive, involving multidisciplinary teams and ongoing funding for curation, policy updates, infrastructure, and community engagement [97]. These substantial costs and logistical complexities continue to hinder widespread clinical integration, underscoring the need for coordinated, well-funded initiatives to translate AI advances into real-world paediatric oncology care.

### Regulatory and ethical constraints

AI in radiology raises questions about transparency, responsibility, and accountability, as regulatory bodies struggle to assess reasoning, predict failures, and define liability, especially when AI detects findings beyond human verification [10, 14]. Professional societies, including the Society for Pediatric Radiology (SPR), the European Society of Paediatric Radiology (ESPR), and the European Society of Radiology and International Society of Paediatric Oncology (SIOP), play a key role in establishing standards, promoting AI literacy, and guiding safe clinical deployment [14, 98, 99]. Nevertheless, AI implementation in healthcare must operate within established legal frameworks, including data protection laws like the General Data Protection Regulation (GDPR) and the Health Insurance Portability and Accountability Act (HIPAA), as well as emerging product safety regulations like the EU Artificial Intelligence Act [12].

AI systems should meet strict quality standards, provide transparent model documentation, and undergo periodic benchmarking. Providers must maintain human oversight to ensure safe and effective clinical use, with clear guidance on intended users and data requirements [10, 98]. Limited and imbalanced paediatric datasets can introduce bias, favouring majority groups or specific institutions [6, 9]. Single-centre studies, retrospective designs, temporal changes, and inconsistent ground-truth further reduce generalisability [6]. Transparency in dataset selection, validation, and reporting is critical to reduce bias and ensure equitable outcomes [13, 83]. Multicentre data harmonisation and standardised evaluation frameworks can help mitigate these risks and improve the reliability and applicability of AI tools in paediatric oncology imaging [11].

## Future perspectives

Radiologists, as well as everyone involved with the use of AI in a clinical setting, require familiarity with AI systems to leverage them safely and effectively in clinical practice. AI excels at processing large datasets and handling repetitive tasks, while radiologists provide judgment, context, and adaptability for complex cases, as well as the essential task of data curation. Combining these strengths can lead to more accurate, efficient, and reliable medical imaging practices, accelerating the translation of AI models into clinical practice [12, 13, 83, 90].

### Overcoming the limitations of paediatric oncology data

The challenges of AI development in paediatric oncology imaging stem from limited data availability and the time-consuming, labour-intensive process of producing annotations, problems exacerbated by the need for datasets diverse enough to represent the entire paediatric age spectrum. These challenges can be addressed in several ways: increasing the availability of data, applying (AI) methods that generate synthetic data or learn effectively from limited examples, or combining both strategies in a complementary manner.

A primary approach to expanding data availability involves multi-institutional collaboration, which can help create datasets that are more diverse across age, demographics, and clinical characteristics. These initiatives rely on standardised protocols and ethical agreements to ensure privacy, reproducibility, and harmonisation [12]. Furthermore, documenting datasets with “datasheets” that detail their composition, collection, and intended uses is critical for responsible implementation [93].

Several dedicated initiatives and platforms now support sharing of paediatric imaging data. Examples of open datasets and online platforms offering downloadable data across a range of disease types, including oncology, are provided in Table 2 and Table 3. These resources can represent a substantial source of labelled and unlabelled data for diverse AI training purposes. Text-based initiatives like the Pediatric Cancer Data Commons [97], which integrate clinical data across tumour types, are particularly valuable. Their impact could be strengthened by coupling them with dedicated imaging databases, creating larger and more diverse datasets for robust AI development. Such paediatric resources are urgently needed, given that large, dedicated imaging datasets have already become indispensable tools for advancing AI research in adult oncology [64, 89, 100].

**Table 2:** Examples of readily downloadable paediatric radiology datasets. This table provides a limited number of examples obtained from public sources, including platforms in Table 3. These open datasets are ready to download but might require the creation of an account on the website or signing of user agreements

Project name	Link download	Type	Label	Image type	Body part
Multi-Consortium International Pediatric Brain tumour Segmentation Challenge 2023 (BraTS-PEDs) [107]	BraTS-PED - syn51514108 - Files [108]	Oncology (glioma)	Labelled (tumour)	MRI	Brain
SynthRAD2023 Grand Challenge [109]	SynthRAD2023 Grand Challenge dataset: synthetising computed tomography for radiotherapy [110]	Oncology (various)	Labelled (patient outline segmentation)	MR, CT, CBCT	Brain, pelvis (paediatric and adult)
HyKid: An Open MRI Dataset with Expert-Annotated Multi-Structure and Choroid Plexus in Pediatric Hydrocephalus [111]	HyKid - syn68544889 - Wiki [112]	Oncology, non oncology	Labelled (brain structures)	MRI	Brain
PediCXR [113]	VinDr-PCXR: an open, large-scale paediatric chest X-ray dataset for interpretation of common thoracic diseases v1.0.0 [114]	Oncology, not oncology	Labelled (various)	X-ray	Chest
Adolescent Brain Cognitive Development (ABCD) Neurocognitive Prediction [115]	NIMH Data Archive - Data - Collection [116]	Not oncology	Not labelled, additional data	MRI	Brain
Autism Brain Imaging Data Exchange [117]	ABIDE [118] and ABIDE [119]	Non oncology	Not labelled, additional data	MRI, various	Brain
Baby Open Brains: An open-source dataset of infant brain segmentations [120]	Baby Open Brains (BOBs) Repository [121]	Non oncology	Labelled (brain structures)	MRI	Brain
Calgary Preschool magnetic resonance imaging (MRI) dataset [122]	OSF | Calgary Preschool MRI Dataset [123]	Non oncology	Not labelled, additional data	MRI	Brain
Child Mind Institute - Healthy Brain Network (HBN) [124]	Data-Portal-Project [125]	Not oncology	Not labelled, additional data	MRI, various	Brain
C-MIND Database [126]	NIMH Data Archive - Data - Collection [127]	Non oncology	Not labelled, additional data	MRI	Brain
Dataset from Power et al. 2012 Neuroimage Sample [128]	Power et al. 2012 Neuroimage Sample [129]	Not oncology	Not labelled, additional data	MRI, various	Brain (paediatric and adult)
Developing Human Connectome Project [130]	Consolidated release, now including data from both foetuses and neonates [131]	Non oncology	Not labelled, additional data	MRI, various	Brain
HEALthy Brain and Child Development (HBCD) Study [132]	Data Tools (ABCD+HBCD) [133]	Non oncology	Not labelled, additional data	MRI, various	Brain
Infancy and early childhood brain MRIs [134]	Large dataset of infancy and early childhood brain MRIs (T1w and T2w) [135]	Non oncology	Not labelled, additional data	MRI	Brain
Neuroimaging dataset on language processing in children ages 5 years, 7 years, and 9 years old [136]	A longitudinal neuroimaging dataset on language processing in children ages 5 years, 7 years, and 9 years old - OpenNeuro [137]	Non oncology	Not labelled, additional data	MRI	Brain
Panoramic Dental X-rays With Segmented Mandibles [138]	Panoramic Dental X-rays With Segmented Mandibles - Mendeley Data [139]	Non oncology	Labelled (mandibles)	X-ray	Mandibles
Pediatric-CT-SEG [140]	PEDIATRIC-CT-SEG - The Cancer Imaging Archive (TCIA) [141]	Non oncology	Labelled (organ contours)	CT, RTSTRUCT	Chest, abdomen, pelvis
PediMS: A Pediatric Multiple Sclerosis Lesion Segmentation Dataset [142]	PediMS: A Pediatric Multiple Sclerosis Lesion Segmentation Dataset [143]	Not oncology	Labelled (lesion segmentation)	MRI	Brain
RSNA Pediatric Bone Age Challenge 2017 [144]	RSNA Bone Age [145]	Not oncology	Labelled (text)	X-Ray	Hand
The Pediatric Imaging, Neurocognition, and Genetics (PING) Data Repository [146]	NIMH Data Archive - Data - Collection [147]	Not oncology	Not labelled, additional data	MRI, various	Brain

*CT* computed tomography, *CBCT* cone beam computed tomography, *MRI* magnetic resonance imaging, *RTSTRUCT* radiotherapy structure sets

**Table 3:** Examples of platforms that host open datasets with focus on paediatric radiological images. In this table is highlighted which platform presents oncological or various other cases (possibly including oncological). The authors suggest search terms such as “cancer”, “tumor”, “tumour”, “pediatric”, “paediatric”, “child”, “children”, “infant”, “adolescent”, “MRI”, “CT”, “segmentation”, “classification”, “mask”, and “label”

Platform name	Link download	Type	Body part
Childhood Cancer Clinical Data Commons (CCDI)	CCDI Hub [148]	Oncology	Various
Children’s Brain tumour Network (CBTN)	Children’s Brain tumour Network [149]	Oncology	Brain
Pediatric Data Collections and Analysis - The Cancer Imaging Archive (TCIA)	Pediatric Data Collections and Analysis - The Cancer Imaging Archive (TCIA) [150]	Oncology	Various
Grand Challenge	Grand Challenge [151]	Oncology, various	Various (paediatric and adult)
Imaging Data Commons (IDC)	IDC [152]	Oncology, various	Various (paediatric and adult)
Kaggle	Find Open Datasets and Machine Learning Projects | Kaggle [153]	Oncology, various	Various (paediatric and adult)
Zenodo	Zenodo [154]	Oncology, various	Various (paediatric and adult)
Institute of Electrical and Electronics Engineers (IEEE) DataPort	Medical Imaging | IEEE DataPort [155]	Various	Various (paediatric and adult)
National Institute of Mental Health Data Archive (NDA, NIMH Data Archive)	NIMH Data Archive - NDA [156]	Various	Brain (paediatric and adult)
NeuroImaging Tools & Resources Collaboratory (NITRC)	NITRC: Welcome [157]	Various	Brain (paediatric and adult)
Radiological Society of North America (RSNA)	AI challenges | RSNA [158]	Various	Various (paediatric and adult)

*CT* computed tomography, *MRI* magnetic resonance imaging

Federated infrastructures, furthermore, enable collaborative AI development across institutions without sharing sensitive patient data, preserving privacy while leveraging heterogeneous, decentralised datasets. This decentralised approach allows models to be trained locally within each participating institution, with only aggregated model updates exchanged across sites [9, 12]. Platforms like the European Federation for Cancer Images (EUCAIM) [101] are pioneering these federated networks, which already include some paediatric data. Access can be tiered, ranging from full data download to restricted on-premises processing where data can be used but never directly viewed or exported. Federated learning has demonstrated performance comparable to traditional centralised training while offering improved data security and scalability [102, 103]. For instance, the FL-PedBrain project, involving 19 international institutions, achieved nearly equivalent accuracy to centralised approaches for paediatric tumour classification and segmentation, underscoring its potential for global paediatric AI collaboration [103].

Beyond increasing data availability, several AI methodologies are specifically designed to mitigate the impact of limited (labelled) data.

**Fig. 3:**
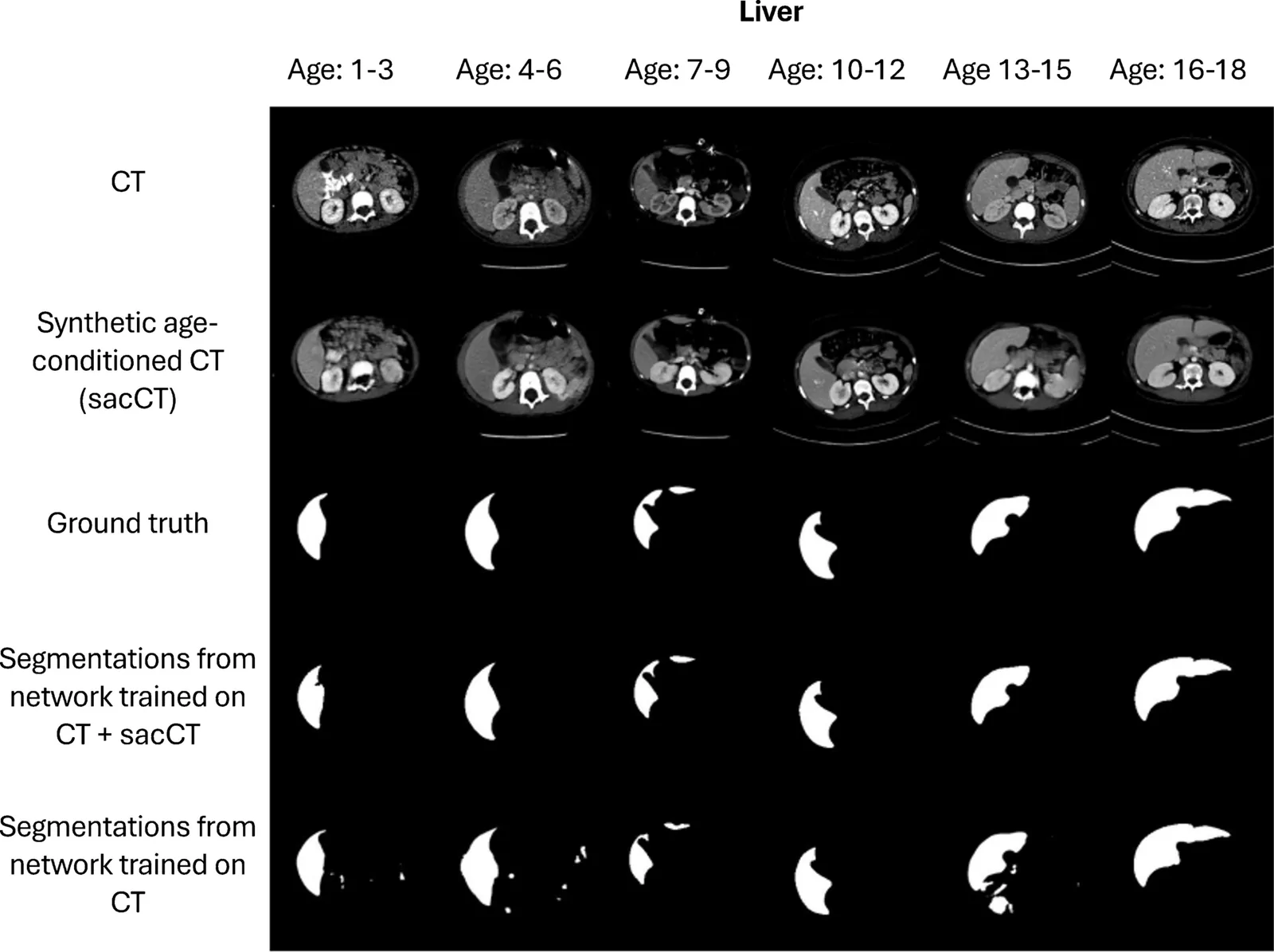
Impact of age-conditioned synthetic data on paediatric liver CT segmentation. Synthetic CT images were generated conditional on patient age, producing segmentation masks that elongate progressively with increasing age to reflect anatomical growth. By capturing this biological variability, the generative model provides effective data augmentation. This enables the segmentation network to learn a more robust representation of paediatric anatomy, ultimately achieving higher accuracy than training on original CT images alone (adapted from Gheshlaghi et al. 2024 [105], “Age Encoded Adversarial Learning for Pediatric CT Segmentation”, Issue 4, Pages 319, Creative Common CC BY license). *CT* computed tomography, *sacCT *synthetic age-conditioned computed tomography

Generative modelling and the use of synthetic data are increasingly employed strategies to reduce reliance on large volumes of paediatric imaging data. They allow the creation of new, potentially labelled artificial images that can be used to train models on a wider variety of examples and to test models in a consistent, repeatable way, all while preserving patient confidentiality [36, 83]. These techniques can generate entirely new scans or modify existing ones by adding or removing lesions (in-painting), often producing images that are indistinguishable from real data [36]. Synthetic datasets can also be tailored to reflect specific demographic or biological factors, helping to balance underrepresented subgroups and fill critical data gaps. For instance, age-conditioned generative models have been used to synthesise paediatric CT images alongside corresponding pancreas segmentation masks, accurately capturing realistic growth trends from infancy through adolescence [104]. These models can also be used to improve downstream tasks such as segmentation; for example, augmenting a paediatric training dataset with synthetic images has been shown to enhance segmentation accuracy for organs like the liver (as demonstrated in Fig. 3), prostate, uterus, and heart [105].

Despite these advantages, generative AI has limitations. Its misuse may inadvertently propagate or amplify existing biases present in the training data, particularly if certain categories are underrepresented. In addition, over-reliance on generated data can also lead models to learn generated features rather than clinically meaningful patterns, limiting their performance on real paediatric images. Consequently, generative AI should not be considered a complete solution to paediatric data scarcity, but rather a complementary approach that requires careful validation and transparent reporting to ensure it alleviates rather than exacerbates existing challenges.

Other strategies, such as self-supervised and weakly supervised learning (Table 1) methods, can help address the scarcity of annotated data, allowing the development of robust models with fewer labelled examples [10, 83]. Furthermore, transfer learning can leverage knowledge from other models (pre-)trained on related (often larger) populations, such as adults with the same disease or patients with similar conditions. This approach can improve performance on specific tasks, especially when the source and target domains share relevant features [162–164]. Some of the most effective frameworks integrate multiple paradigms; for instance, a paediatric chest X-ray framework combining self-supervised pre-training with transfer learning from adult datasets achieved performance comparable to fully supervised models while requiring ten times fewer labelled examples [92].

An important note is that AI methods may widely vary in terms of data requirements based on their complexity and the tasks to learn. Broadly, two families of methods can be identified: task-specific models versus generalist foundation models. On the one hand, task-specific models aim to solve a well-defined problem. This kind of model has a relatively simple architecture (e.g. less parameters) for nowadays AI-standards. As such, task-specific models tend to be more interpretable, easier to implement and monitor within clinical workflows, and they may not require large amounts of data and high-end hardware to be successfully trained [13]. However, a disadvantage of these models is that they address isolated tasks and may not be interoperable, often requiring a suite of tools to cover the full radiological workflow.

On the other hand, FMs (Table 1) are large models trained on extensive generic datasets, making them generally well-suited for transfer learning with minimal training examples (few-shot learning). Their large-scale pre-training allows them to adapt to new tasks with relatively less additional task-specific paediatric data. This can include patient-specific adaptations by using only a few examples per patient [106], representing a potential step toward personalised medicine. However, FMs come with notable challenges: while they can be typically fine-tuned for many tasks, their performance on highly specialised paediatric tasks with limited data is not guaranteed. If complete re-training is needed, this typically requires data sizes and computational resources that might not be commonly available. Furthermore, their outputs must be carefully validated for clinical safety due to their opaque decision-making processes, which might be a more complex challenge than with smaller models. Additionally, the scale of their pre-training makes it challenging to document the origin of their training data, a critical safeguard to prevent their application on incorrect populations.

Ultimately, the choice between smaller, task-specific models and large FMs depends on the clinical problem at hand. While FMs promise greater flexibility and the potential to perform multiple tasks within a single architecture, smaller models currently provide significant benefits in interpretability, resource efficiency, and immediate clinical integration. Until data scarcity in paediatric oncology imaging is more robustly addressed, the field will likely rely on a suite of specialised, validated tools, as no single program can yet replicate a clinician’s broad interpretive capacity [13, 14].

### Steps toward safe adoption of artificial intelligence in paediatric oncology

Before AI can be safely and effectively deployed in paediatric oncology, clinicians and radiologists require a thorough understanding of its capabilities and its limitations, a foundation that rests on achieving AI literacy [14]. Ensuring that AI models are explainable and trustworthy is essential for clinical adoption, particularly in the sensitive field of paediatrics. A significant barrier to this understanding is that many AI models function as “black boxes”, producing predictions without a clear explanation of their reasoning, which can reduce clinician confidence and hinder integration into healthcare [9, 12, 64].

Explainable AI (XAI) addresses this by providing methods to clarify a model’s decision-making process. These techniques are categorised by when they are applied. Pre-model interpretability focuses on analysing and visualising the input data itself before any model is built. In-model interpretability involves designing models that are inherently transparent, where the reasoning process is built into their structure. Finally, post-model interpretability uses external methods to explain the predictions of already-trained, complex “black-box” models without altering their internal workings. Together, these techniques allow both local explanations for single predictions and global insights into model behaviour, improving clinician trust and enabling research validation [63]. However, explainable AI alone does not guarantee clinical reliability. Trustworthy AI integrates interpretability with principles of robustness, fairness, and privacy, ensuring that models remain safe and ethically aligned across diverse populations and imaging settings [63]. Furthermore, rigorous performance evaluation of each application and for every indication is needed, as single metrics like accuracy are insufficient to capture clinical utility [159].

Ultimately, the successful adoption of these technologies necessitates foundational education. The implementation of AI education in the medical, paediatric oncology and radiology curricula will be an important cornerstone in achieving sufficient knowledge to understand the basics of AI, which in turn will lead to a better understanding and collaboration between clinicians and AI developers [160, 161].

## Conclusion

Paediatric oncology imaging could benefit immensely from the implementation of AI, yet the lack of paediatric-specific tools and data availability hinders research and clinical implementation. Addressing this gap requires both data-oriented strategies, such as open datasets, federated learning, or multi-institute collaboration, as well as AI-oriented strategies, like transfer learning, generative modelling, and foundation models to maximise the potential of limited data. Together, these can create more diverse and robust resources, enabling AI to support clinicians across the oncologic pipeline, from acquisition and preprocessing to detection, diagnosis, staging, prognosis, monitoring, and reporting. Achieving this, however, demands not only technical advances but also sustained investment, careful validation, explainability, and strong collaboration between clinicians, data scientists, and regulatory bodies to ensure safe and equitable implementation in paediatric oncology.

## Supplementary Information

Below is the link to the electronic supplementary material.

ESM 1 Supplementary Material 1 (PDF 190 KB)

ESM 2 Supplementary Material 2 (PDF 37.4 KB)

## Data Availability

No datasets were generated or analysed during the current study.
