# Supplementary material for: Application of artificial intelligence in paediatric oncology imaging
Source: Pediatr Radiol. 2026 Apr 28;56(9):1975–95. doi: 10.1007/s00247-026-06606-1 (PMC13546343; doi:10.1007/s00247-026-06606-1)
Supplement: Supplementary file 1 [file 247_2026_6606_MOESM1_ESM.pdf]

Supplementary Material 1.
Overview of methodology and patient population in the referenced publications

Reference	Type of research	Adult	Paediatric	Not specified / Not applicable[a]
Afshari Mirak et al. 2025 [4]	Report			x
Alam et al. 2024 [53]	Systematic review			x
Ankush. 2025 [86]	Original research			x
Ardahan Sevgili and Şenol. 2023 [81]	Scoping review		x	
Bani-Ahmad et al. 2025 [19]	Scoping review	x	x	
Benitez-Aurioles et al. 2025 [29]	Original research		x	
Bhattacharya et al. 2023 [38]	Original research		x	
Bhayana et al. 2025 [82]	Original research	x		
Boellaard et al. 2024 [69]	Original research			x
Boyd et al. 2024 [90]	Original research		x	
Buser et al. 2025[56]	Original research		x	
Buser et al. 2025 [91]	Scoping review		x	
Chatterjee et al. 2025 [68]	Original research	x	x	
Chatziantoniou et al. 2024 [43]	Original research		x	
Cheng et al. 2024 [92]	Original research		x	
Chupetlovska et al. 2025 [61]	Report			x
Clement et al. 2025 [18]	Scoping review			x
Dalboni da Rocha et al. 2025 [12]	Scoping review		x	

De Luca et al. 2025 [45]	Original research	x		
di Noia et al. 2022 [74]	Scoping review			x
Ding et al. 2025 [55]	Original research		x	
Enoch Kan et al. 2020 [104]	Original research		x	
Familiar et al. 2024 [59]	Scoping review		x	
Farmakis et al. 2021 [5]	Report		x	
Fitski et al. 2020 [57]	Original research		x	
Florkow et al. 2020 [34]	Original research		x	
Ganatra. 2025 [9]	Scoping review		x	x
Gao et al. 2025 [62]	Scoping review			x
Gebru et al. 2021 [93]	Original research			x
Gheshlaghi et al. 2024 [105]	Original research		x	
Gong et al. 2018 [25]	Original research	x		
Gotkowski et al. 2025 [65]	Scoping review			x
Grist et al. 2021 [75]	Original research		x	
Hassan et al. 2025 [17]	Scoping review		x	
Hernandez-Cruz et al. 2024 [102]	Scoping review			x
Hosny et al. 2025 [10]	Original data			x
Hua et al. 2025 [89]	Scoping review	x	x	
Huang et al. 2023 [54]	Scoping review			x
Huang et al. 2021 [47]	Original research			x
Hui et al. 2025 [160]	Scoping review			x
Isensee et al. 2018 [15]	Original research			x
Johnson et al. 2025 [88]	Report		x	
Kann et al. 2025 [77]	Scoping review		x	
Kazerooni et al. 2025 [78]	Scoping review		x	
Kelly et al. 2024 [6]	Scoping review		x	

Khosravi et al. 2024 [24]	Scoping review			x
Kim et al. 2022 [162]	Scoping review			x
Klontzas et al. 2025 [159]	Scoping review			x
Kotter et al. 2025 [98]	Report			x
Lee et al. 2024 [103]	Original research		x	
Ma et al. 2024 [67]	Original research			x
Mahootiha et al. 2025 [80]	Original research		x	
Martí-Bonmati et al. 2025 [101]	Report			x
Matute-González et al. 2024 [84]	Original research			x
McKenzie et al. 2020 [51]	Original research			x
van der Mee et al. 2025 [87]	Systematic review			x
Meshaka et al. 2023 [31]	Scoping review		x	
Misera et al. 2024 [163]	Scoping review			x
Mohammadinejad et al. 2021 [21]	Scoping review			x
Montgomery et al. 2023 [37]	Original research			x
Montin et al. 2020 [52]	Original research		x	
Nagayama et al. 2021 [23]	Scoping review		x	
Nagaraj et al. 2025 [39]	Original research		x	
Ng. 2022 [22]	Systematic review		x	
Ng. 2023 [40]	Systematic review		x	
Nikam et al. 2022 [48]	Scoping review		x	
Nievelstein et al. 2025 [70]	Scoping review		x	
Noh and Lee. 2025 [164]	Scoping review			x
Obuchowicz et al. 2025 [13]	Scoping review			x
Otjen et al. 2022 [14]	Scoping review		x	
Pacchiano et al. 2024 [60]	Scoping review		x	
Pachetti and Colantonio. 2024 [106]	Systematic review			x

Peretti et al. 2023 [165]	Original research			x
Pfaehler et al. 2022 [166]	Original research			x
Raut et al. 2024 [41]	Original research			x
Sacoransky et al. 2024 [85]	Scoping review			x
Seah et al. 2025 [8]	Report			x
Seoni et al. 2024 [11]	Systematic review			x
Shafighi Shahri et al. 2025 [64]	Systematic review		x	
Sherwani and Gopalakrishna. 2024 [33]	Systematic review			x
Singh et al. 2020 [100]	Original research		x	
Singh et al. 2024 [28]	Scoping review	x	x	
Sizikova et al. 2024 [36]	Scoping review	x	x	
Skaarup et al. 2021 [49]	Original research		x	
Tak et al. 2025 [79]	Original research		x	
Teng et al. 2024 [63]	Scoping review			x
Uus et al. 2023 [32]	Report		x	
Valls-Esteve et al. 2023 [50]	Case reports		x	
Verghese et al. 2025 [161]	Scoping review			x
Vinit et al. 2024 [58]	Scoping review		x	
Vymazal and Rulesh. 2024 [26]	Scoping review			x
Wasserthal et al. 2023 [16]	Original research	x		
Wiestler et al. 2024 [71]	Original research		x	
Zhang et al. 2022 [42]	Original research		x	
Zhang et al. 2023 [76]	Scoping review			x
Zhang et al. 2024 [27]	Original research		x	
Zhao et al. 2019 [20]	Original research			x
Zhou et al. 2024 [30]	Scoping review			x

[a] The authors did not describe the included patient population in sufficient detail.

References

1. Steliarova-Foucher E, Colombet M, Ries LAG, et al (2017) International incidence of childhood cancer, 2001–10: a population-based registry study. Lancet Oncol 18:719–731. https://doi.org/10.1016/S1470-2045(17)30186-9
2. Wang CM, Whiting AH, Rath A, et al (2024) Operational description of rare diseases: a reference to improve the recognition and visibility of rare diseases. Orphanet J Rare Dis 19:334. https://doi.org/10.1186/s13023-024-03322-7
3. Reekers JA (2014) The spider model for clinical involvement in radiology. Insights Imaging 5:403–405. https://doi.org/10.1007/s13244-014-0326-4
4. Afshari Mirak S, Tirumani SH, Ramaiya N, Mohamed I (2025) The Growing Nationwide Radiologist Shortage: Current Opportunities and Ongoing Challenges for International Medical Graduate Radiologists. Radiology 314:e232625. https://doi.org/10.1148/radiol.232625
5. Farmakis SG, Chertoff JD, Barth RA (2021) Pediatric Radiologist Workforce Shortage: Action Steps to Resolve. J Am Coll Radiol 18:1675–1677. https://doi.org/10.1016/j.jacr.2021.07.026
6. Kelly BS, Clifford SM, Judge CS, et al (2024) A survey of Paediatric Radiology Artificial Intelligence. 2024.09.19.24313885
7. Morales-Tisnés T, Pelzl CE, Christensen EW, et al (2025) Defining the Pediatric Radiology Workforce in the United States Using Insurance Claims Data. J Am Coll Radiol S154614402500448X. https://doi.org/10.1016/j.jacr.2025.07.029
8. Seah JCY, Tang JSN, Tran A (2025) Drafting the Future: The Dawn of AI Report Generation in Radiology. Radiology 316:e243378. https://doi.org/10.1148/radiol.243378
9. Ganatra HA (2025) Machine Learning in Pediatric Healthcare: Current Trends, Challenges, and Future Directions. J Clin Med 14:807. https://doi.org/10.3390/jcm14030807
10. Hosny A, Parmar C, Quackenbush J, et al (2018) Artificial intelligence in radiology. Nat Rev Cancer 18:500–510. https://doi.org/10.1038/s41568-018-0016-5
11. Seoni S, Shahini A, Meiburger KM, et al (2024) All you need is data preparation: A systematic review of image harmonization techniques in Multi-center/device studies for medical support systems. Comput Methods Programs Biomed 250:108200. https://doi.org/10.1016/j.cmpb.2024.108200
12. Dalboni da Rocha JL, Lai J, Pandey P, et al (2025) Artificial Intelligence for Neuroimaging in Pediatric Cancer. Cancers 17:622. https://doi.org/10.3390/cancers17040622
13. Obuchowicz R, Lasek J, Wodziński M, et al (2025) Artificial Intelligence-Empowered Radiology—Current Status and Critical Review. Diagnostics 15:282. https://doi.org/10.3390/diagnostics15030282
14. Otjen JP, Moore MM, Romberg EK, et al (2022) The current and future roles of artificial intelligence in pediatric radiology. Pediatr Radiol 52:2065–2073. https://doi.org/10.1007/s00247-021-05086-9
15. Isensee F, Petersen J, Klein A, et al (2018) nnU-Net: Self-adapting Framework for U-Net-Based Medical Image Segmentation
16. Wasserthal J, Breit H-C, Meyer MT, et al (2023) TotalSegmentator: Robust Segmentation of 104 Anatomic Structures in CT Images. Radiol Artif Intell 5:e230024. https://doi.org/10.1148/ryai.230024

17. Hassan M, Shahzadi S, Kloczkowski A (2025) Harnessing Artificial Intelligence in Pediatric Oncology Diagnosis and Treatment: A Review. Cancers 17:1828. https://doi.org/10.3390/cancers17111828
18. Clement David-Olawade A, Olawade DB, Vanderbloemen L, et al (2025) AI-Driven Advances in Low-Dose Imaging and Enhancement—A Review. Diagnostics 15:689. https://doi.org/10.3390/diagnostics15060689
19. Bani-Ahmad M, England A, McLaughlin L, et al (2025) Potential of artificial intelligence for radiation dose reduction in computed tomography —A scoping review. Radiography 31:102968. https://doi.org/10.1016/j.radi.2025.102968
20. Zhao C, Shao M, Carass A, et al (2019) Applications of a deep learning method for anti-aliasing and super-resolution in MRI. Magn Reson Imaging 64:132–141. https://doi.org/10.1016/j.mri.2019.05.038
21. Mohammadinejad P, Mileto A, Yu L, et al (2021) CT Noise-Reduction Methods for Lower-Dose Scanning: Strengths and Weaknesses of Iterative Reconstruction Algorithms and New Techniques. Radiogr Rev Publ Radiol Soc N Am Inc 41:1493–1508. https://doi.org/10.1148/rg.2021200196
22. Ng CKC (2022) Artificial Intelligence for Radiation Dose Optimization in Pediatric Radiology: A Systematic Review. Children 9:1044. https://doi.org/10.3390/children9071044
23. Nagayama Y, Sakabe D, Goto M, et al (2021) Deep Learning–based Reconstruction for Lower-Dose Pediatric CT: Technical Principles, Image Characteristics, and Clinical Implementations. RadioGraphics 41:1936–1953. https://doi.org/10.1148/rg.2021210105
24. Khosravi P, Mohammadi S, Zahiri F, et al (2024) AI-Enhanced Detection of Clinically Relevant Structural and Functional Anomalies in MRI: Traversing the Landscape of Conventional to Explainable Approaches. J Magn Reson Imaging 60:2272–2289. https://doi.org/10.1002/jmri.29247
25. Gong E, Pauly JM, Wintermark M, Zaharchuk G (2018) Deep learning enables reduced gadolinium dose for contrast-enhanced brain MRI. J Magn Reson Imaging JMRI 48:330–340. https://doi.org/10.1002/jmri.25970
26. Vymazal J, Rulseh AM (2024) MRI contrast agents and retention in the brain: review of contemporary knowledge and recommendations to the future. Insights Imaging 15:179. https://doi.org/10.1186/s13244-024-01763-z
27. Zhang Q, Hu Y, Zhou C, et al (2024) Reducing pediatric total-body PET/CT imaging scan time with multimodal artificial intelligence technology. EJNMMI Phys 11:1. https://doi.org/10.1186/s40658-023-00605-z
28. Singh SB, Sarrami AH, Gatidis S, et al (2024) Applications of Artificial Intelligence for Pediatric Cancer Imaging. Am J Roentgenol 223:. https://doi.org/10.2214/ajr.24.31076
29. Benitez-Aurioles J, Osorio EMV, Aznar MC, et al (2025) A neural network to create super-resolution MR from multiple 2D brain scans of pediatric patients. Med Phys 52:1693–1705. https://doi.org/10.1002/mp.17563
30. Zhou Z, Hu P, Qi H (2024) Stop moving: MR motion correction as an opportunity for artificial intelligence. Magn Reson Mater Phys Biol Med 37:397–409. https://doi.org/10.1007/s10334-023-01144-5
31. Meshaka R, Gaunt T, Shelmerdine SC (2023) Artificial intelligence applied to fetal MRI: A scoping review of current research. Br J Radiol 96:20211205. https://doi.org/10.1259/bjr.20211205

32. Uus AU, Egloff Collado A, Roberts TA, et al (2023) Retrospective motion correction in foetal MRI for clinical applications: existing methods, applications and integration into clinical practice. Br J Radiol 96:20220071. https://doi.org/10.1259/bjr.20220071
33. Sherwani MK, Gopalakrishnan S (2024) A systematic literature review: deep learning techniques for synthetic medical image generation and their applications in radiotherapy. Front Radiol 4:. https://doi.org/10.3389/fradi.2024.1385742
34. Florkow MC, Guerreiro F, Zijlstra F, et al (2020) Deep learning-enabled MRI-only photon and proton therapy treatment planning for paediatric abdominal tumours. Radiother Oncol 153:220–227. https://doi.org/10.1016/j.radonc.2020.09.056
35. Kleinerman RA (2009) Radiation-sensitive genetically susceptible pediatric sub-populations. Pediatr Radiol 39 Suppl 1:S27-31. https://doi.org/10.1007/s00247-008-1015-6
36. Sizikova E, Badal A, Delfino JG, et al (2024) Synthetic data in radiological imaging: current state and future outlook. BJRArtificial Intell 1:ubae007. https://doi.org/10.1093/bjrai/ubae007
37. Montgomery ME, Andersen FL, d'Este SH, et al (2023) Attenuation Correction of Long Axial Field-of-View Positron Emission Tomography Using Synthetic Computed Tomography Derived from the Emission Data: Application to Low-Count Studies and Multiple Tracers. Diagnostics 13:3661. https://doi.org/10.3390/diagnostics13243661
38. Bhattacharya S, Bhattacharya A, Shahnawaz S (2023) Generating Synthetic Computed Tomography (CT) Images to Improve the Performance of Machine Learning Model for Pediatric Abdominal Anomaly Detection. In: 2023 IEEE/CVF International Conference on Computer Vision Workshops (ICCVW). IEEE, Paris, France, pp 3867–3875
39. Nagaraj UD, Meineke J, Sriwastwa A, et al (2025) Evaluation of synthetic images derived from a neural network in pediatric brain magnetic resonance imaging. Magn Reson Imaging 121:110427. https://doi.org/10.1016/j.mri.2025.110427
40. Ng CKC (2023) Generative Adversarial Network (Generative Artificial Intelligence) in Pediatric Radiology: A Systematic Review. Children 10:1372. https://doi.org/10.3390/children10081372
41. Raut P, Baldini G, Schöneck M, Caldeira L (2024) Using a generative adversarial network to generate synthetic MRI images for multi-class automatic segmentation of brain tumors. Front Radiol 3:. https://doi.org/10.3389/fradi.2023.1336902
42. Zhang H, Li H, Dillman JR, et al (2022) Multi-Contrast MRI Image Synthesis Using Switchable Cycle-Consistent Generative Adversarial Networks. Diagnostics 12:816. https://doi.org/10.3390/diagnostics12040816
43. Chatziantoniou C, van Ewijk R, Adams M, et al (2024) Acquisition matters - How do scan parameters affect ADC estimates in pediatric rhabdomyosarcoma?
44. Boellaard R, Delgado-Bolton R, Oyen WJG, et al (2015) FDG PET/CT: EANM procedure guidelines for tumour imaging: version 2.0. Eur J Nucl Med Mol Imaging 42:328–354. https://doi.org/10.1007/s00259-014-2961-x
45. De Luca A, Swartenbroekx T, Seelaar H, et al (2025) Cross-site harmonization of diffusion MRI data without matched training subjects. Magn Reson Med 94:1750–1762. https://doi.org/10.1002/mrm.30575
46. van Ewijk R, Schoot RA, Sparber-Sauer M, et al (2021) European guideline for imaging in paediatric and adolescent rhabdomyosarcoma — joint statement by the European Paediatric Soft Tissue Sarcoma Study Group, the Cooperative Weichteilsarkom

Studiengruppe and the Oncology Task Force of the European Society of Paediatric Radiology. Pediatr Radiol 51:1940–1951. https://doi.org/10.1007/s00247-021-05081-0
47. Huang Y, Ahmad S, Fan J, et al (2021) Difficulty-aware hierarchical CNNs for deformable registration of brain MR images. Med Image Anal 67:101817. https://doi.org/10.1016/j.media.2020.101817
48. Nikam RM, Yue X, Kaur G, et al (2022) Advanced Neuroimaging Approaches to Pediatric Brain Tumors. Cancers 14:3401. https://doi.org/10.3390/cancers14143401
49. Skaarup M, Lundemann MJ, Darkner S, et al (2021) A framework for voxel-based assessment of biological effect after proton radiotherapy in pediatric brain cancer patients using multi-modal imaging. Med Phys 48:4110–4121. https://doi.org/10.1002/mp.14989
50. Valls-Esteve A, Adell-Gómez N, Pasten A, et al (2023) Exploring the Potential of Three-Dimensional Imaging, Printing, and Modeling in Pediatric Surgical Oncology: A New Era of Precision Surgery. Children 10:832. https://doi.org/10.3390/children10050832
51. McKenzie EM, Santhanam A, Ruan D, et al (2020) Multimodality image registration in the head-and-neck using a deep learning-derived synthetic CT as a bridge. Med Phys 47:1094–1104. https://doi.org/10.1002/mp.13976
52. Montin E, Belfatto A, Bologna M, et al (2020) A multi-metric registration strategy for the alignment of longitudinal brain images in pediatric oncology. Med Biol Eng Comput 58:843–855. https://doi.org/10.1007/s11517-019-02109-4
53. Alam MA, Amir Sohel, Hasan KM, Ahmad I (2024) Advancing Brain Tumor Detection Using Machine Learning And Artificial Intelligence: A Systematic Literature Review Of Predictive Models And Diagnostic Accuracy. Strateg Data Manag Innov 1:37–55. https://doi.org/10.71292/sdmi.v1i01.6
54. Huang S, Yang J, Shen N, et al (2023) Artificial intelligence in lung cancer diagnosis and prognosis: Current application and future perspective. Semin Cancer Biol 89:30–37. https://doi.org/10.1016/j.semcancer.2023.01.006
55. Ding M, Maspero M, Littooij AS, et al (2025) Deep learning-based auto-contouring of organs/structures-at-risk for pediatric upper abdominal radiotherapy. Radiother Oncol 208:110914. https://doi.org/10.1016/j.radonc.2025.110914
56. Buser MAD, Simons DC, Fitski M, et al (2025) A Community Benchmark for the Automated Segmentation of Pediatric Neuroblastoma on Multi-Modal MRI: Design and Results of the SPPIN Challenge at MICCAI 2023. Bioengineering 12:1157. https://doi.org/10.3390/bioengineering12111157
57. Fitski M, Meulstee JW, Littooij AS, et al (2020) MRI-Based 3-Dimensional Visualization Workflow for the Preoperative Planning of Nephron-Sparing Surgery in Wilms' Tumor Surgery: A Pilot Study. J Healthc Eng 2020:8899049. https://doi.org/10.1155/2020/8899049
58. Vinit N, Blanc T, Bloch I, et al (2024) Robotics and 3D modeling for precision surgery in pediatric oncology. EJC Paediatr Oncol 4:100181. https://doi.org/10.1016/j.ejcped.2024.100181
59. Familiar AM, Fathi Kazerooni A, Vossough A, et al (2024) Towards consistency in pediatric brain tumor measurements: Challenges, solutions, and the role of artificial intelligence-based segmentation. Neuro-Oncol 26:1557–1571. https://doi.org/10.1093/neuonc/noae093
60. Pacchiano F, Tortora M, Doneda C, et al (2024) Radiomics and artificial intelligence applications in pediatric brain tumors. World J Pediatr 20:747–763. https://doi.org/10.1007/s12519-024-00823-0

61. Chupetlovska K, Akinci D'Antonoli T, Bodalal Z, et al (2025) ESR Essentials: a step-by-step guide of segmentation for radiologists-practice recommendations by the European Society of Medical Imaging Informatics. Eur Radiol 35:6894–6904. https://doi.org/10.1007/s00330-025-11621-1
62. Gao Y, Jiang Y, Peng Y, et al (2025) Medical Image Segmentation: A Comprehensive Review of Deep Learning-Based Methods. Tomography 11:52. https://doi.org/10.3390/tomography11050052
63. Teng Z, Li L, Xin Z, et al (2024) A literature review of artificial intelligence (AI) for medical image segmentation: from AI and explainable AI to trustworthy AI. Quant Imaging Med Surg 14:9620–9652. https://doi.org/10.21037/qims-24-723
64. Shafighi Shahri E, Ehsasatvatan A, Salavatizadeh M, Talaeepur S (2025) Artificial Intelligence Applications in Pediatric Oncology Imaging: A Systematic Review of Diagnostic, Prognostic, and Therapeutic Evaluation Tools. Infosci Trends 2:36–47. https://doi.org/10.61186/ist.202502.03.04
65. Gotkowski K, Maier-Hein KH, Isensee F (2025) Revisiting 3D Medical Scribble Supervision: Benchmarking Beyond Cardiac Segmentation
66. Ronneberger O, Fischer P, Brox T (2015) U-Net: Convolutional Networks for Biomedical Image Segmentation
67. Ma J, He Y, Li F, et al (2024) Segment anything in medical images. Nat Commun 15:654. https://doi.org/10.1038/s41467-024-44824-z
68. Chatterjee D, Kanhere A, Doo FX, et al (2025) Children Are Not Small Adults: Addressing Limited Generalizability of an Adult Deep Learning CT Organ Segmentation Model to the Pediatric Population. J Imaging Inform Med 38:1628–1641. https://doi.org/10.1007/s10278-024-01273-w
69. Boellaard R, Buvat I, Nioche C, et al (2024) International Benchmark for Total Metabolic Tumor Volume Measurement in Baseline 18F-FDG PET/CT of Lymphoma Patients: A Milestone Toward Clinical Implementation. J Nucl Med Off Publ Soc Nucl Med 65:1343–1348. https://doi.org/10.2967/jnumed.124.267789
70. Nievelstein RAJ, Borgwardt L, Inarejos Clemente EJ, et al (2025) White paper on the current state of imaging biomarkers in paediatric oncology. Pediatr Radiol. https://doi.org/10.1007/s00247-025-06372-6
71. Wiestler B, Bison B, Behrens L, et al (2024) Human-Level Differentiation of Medulloblastoma from Pilocytic Astrocytoma: A Real-World Multicenter Pilot Study. Cancers 16:1474. https://doi.org/10.3390/cancers16081474
72. Eisenhauer EA, Therasse P, Bogaerts J, et al (2009) New response evaluation criteria in solid tumours: revised RECIST guideline (version 1.1). Eur J Cancer Oxf Engl 1990 45:228–247. https://doi.org/10.1016/j.ejca.2008.10.026
73. Miller AB, Hoogstraten B, Staquet M, Winkler A (1981) Reporting results of cancer treatment. Cancer 47:207–214. https://doi.org/10.1002/1097-0142(19810101)47:1%253C207::AID-CNCR2820470134%253E3.0.CO;2-6
74. di Noia C, Grist JT, Riemer F, et al (2022) Predicting Survival in Patients with Brain Tumors: Current State-of-the-Art of AI Methods Applied to MRI. Diagnostics 12:2125. https://doi.org/10.3390/diagnostics12092125
75. Grist JT, Withey S, Bennett C, et al (2021) Combining multi-site magnetic resonance imaging with machine learning predicts survival in pediatric brain tumors. Sci Rep 11:18897. https://doi.org/10.1038/s41598-021-96189-8

76. Zhang B, Shi H, Wang H (2023) Machine Learning and AI in Cancer Prognosis, Prediction, and Treatment Selection: A Critical Approach. J Multidiscip Healthc 16:1779–1791. https://doi.org/10.2147/JMDH.S410301
77. Kann BH, Vossough A, Brüningk SC, et al (2025) Artificial Intelligence for Response Assessment in Pediatric Neuro-Oncology (AI-RAPNO), part 1: review of the current state of the art. Lancet Oncol 26:e597–e606. https://doi.org/10.1016/S1470-2045(25)00484-X
78. Kazerooni AF, Familiar AM, Aboian M, et al (2025) Artificial Intelligence for Response Assessment in Pediatric Neuro-Oncology (AI-RAPNO), part 2: challenges, opportunities, and recommendations for clinical translation. Lancet Oncol 26:e607–e618. https://doi.org/10.1016/S1470-2045(25)00489-9
79. Tak D, Garomsa BA, Zapaishchykova A, et al (2025) Longitudinal Risk Prediction for Pediatric Glioma with Temporal Deep Learning. NEJM AI 2:AIoa2400703. https://doi.org/10.1056/AIoa2400703
80. Mahootiha M, Tak D, Ye Z, et al (2025) Multimodal deep learning improves recurrence risk prediction in pediatric low-grade gliomas. Neuro-Oncol 27:277–290. https://doi.org/10.1093/neuonc/noae173
81. Ardahan Sevgili S, Şenol S (2023) Prediction of chemotherapy-related complications in pediatric oncology patients: artificial intelligence and machine learning implementations. Pediatr Res 93:390–395. https://doi.org/10.1038/s41390-022-02356-6
82. Bhayana R, Alwahbi O, Ladak AM, et al (2025) Leveraging Large Language Models to Generate Clinical Histories for Oncologic Imaging Requisitions. Radiology 314:e242134. https://doi.org/10.1148/radiol.242134
83. Paschali M, Chen Z, Blankemeier L, et al (2025) Foundation Models in Radiology: What, How, Why, and Why Not. Radiology 314:e240597. https://doi.org/10.1148/radiol.240597
84. Matute-González M, Darnell A, Comas-Cufí M, et al (2024) Utilizing a domain-specific large language model for LI-RADS v2018 categorization of free-text MRI reports: a feasibility study. Insights Imaging 15:280. https://doi.org/10.1186/s13244-024-01850-1
85. Sacoransky E, Kwan BYM, Soboleski D (2024) ChatGPT and assistive AI in structured radiology reporting: A systematic review. Curr Probl Diagn Radiol 53:728–737. https://doi.org/10.1067/j.cpradiol.2024.07.007
86. Ankush A (2025) VoxRad: Building an open-source locally-hosted radiology reporting system. Clin Imaging 119:110414. https://doi.org/10.1016/j.clinimag.2025.110414
87. van der Mee F a. M, Ottenheijm RPG, Gentry EGS, et al (2025) The impact of different radiology report formats on patient information processing: a systematic review. Eur Radiol 35:2644–2657. https://doi.org/10.1007/s00330-024-11165-w
88. Johnson KB, Simonian M, Adams LL, Schneider JH (2025) Toward Trustworthy Pediatric AI: A Call to Action From the National Academy of Medicine. Pediatrics 156:e2025073304. https://doi.org/10.1542/peds.2025-073304
89. Hua SBZ, Heller N, He P, et al (2025) Lack of children in public medical imaging data points to growing age bias in biomedical AI. MedRxiv Prepr Serv Health Sci 2025.06.06.25328913. https://doi.org/10.1101/2025.06.06.25328913
90. Boyd A, Ye Z, Prabhu SP, et al (2024) Stepwise Transfer Learning for Expert-level Pediatric Brain Tumor MRI Segmentation in a Limited Data Scenario. Radiol Artif Intell 6:e230254. https://doi.org/10.1148/ryai.230254

91. Buser MAD, van der Rest JK, Wijnen MHWA, et al (2025) Deep Learning and Multidisciplinary Imaging in Pediatric Surgical Oncology: A Scoping Review. Cancer Med 14:e70574. https://doi.org/10.1002/cam4.70574
92. Cheng S, Starosolski ZA, Subramanian D (2024) Self-Supervised Learning for Building Robust Pediatric Chest X-ray Classification Models
93. Gebru T, Morgenstern J, Vecchione B, et al (2021) Datasheets for Datasets
94. Berkhout WEM, van Wijngaarden JJ, Workum JD, et al (2025) Operationalization of Artificial Intelligence Applications in the Intensive Care Unit: A Systematic Review. JAMA Netw Open 8:e2522866. https://doi.org/10.1001/jamanetworkopen.2025.22866
95. Products. In: Health AI Regist. https://healthairegister.com/radiology/products. Accessed 24 Jan 2026
96. AI Landscape. https://aicentral.acrdsi.org//AI-Landscape. Accessed 24 Jan 2026
97. Plana A, Furner B, Palese M, et al (2021) Pediatric Cancer Data Commons: Federating and Democratizing Data for Childhood Cancer Research. JCO Clin Cancer Inform 1034–1043. https://doi.org/10.1200/CCI.21.00075
98. Kotter E, D'Antonoli TA, Cuocolo R, et al (2025) Guiding AI in radiology: ESR's recommendations for effective implementation of the European AI Act. Insights Imaging 16:33. https://doi.org/10.1186/s13244-025-01905-x
99. Shelmerdine SC, Naidoo J, Kelly BS, et al (2025) AI implementation in pediatric radiology for patient safety: a multi-society statement from the ACR, ESPR, SPR, SLARP, AOSPR, SPIN. Pediatr Radiol. https://doi.org/10.1007/s00247-025-06386-0
100. Singh A, Salehi SSM, Gholipour A (2020) Deep Predictive Motion Tracking in Magnetic Resonance Imaging: Application to Fetal Imaging. IEEE Trans Med Imaging 39:3523–3534. https://doi.org/10.1109/TMI.2020.2998600
101. Martí-Bonmatí L, Blanquer I, Tsiknakis M, et al (2025) Empowering cancer research in Europe: the EUCAIM cancer imaging infrastructure. Insights Imaging 16:47. https://doi.org/10.1186/s13244-025-01913-x
102. Hernandez-Cruz N, Saha P, Sarker MMK, Noble JA (2024) Review of Federated Learning and Machine Learning-Based Methods for Medical Image Analysis. Big Data Cogn Comput 8:99. https://doi.org/10.3390/bdcc8090099
103. Lee EH, Han M, Wright J, et al (2024) An international study presenting a federated learning AI platform for pediatric brain tumors. Nat Commun 15:7615. https://doi.org/10.1038/s41467-024-51172-5
104. Enoch Kan CN, Maheenaboobacker N, Ye DH (2020) Age-Conditioned Synthesis of Pediatric Computed Tomography with Auxiliary Classifier Generative Adversarial Networks. In: 2020 IEEE 17th International Symposium on Biomedical Imaging (ISBI). IEEE, Iowa City, IA, USA, pp 109–112
105. Gheshlaghi SH, Kan CNE, Schmidt TG, Ye DH (2024) Age Encoded Adversarial Learning for Pediatric CT Segmentation. Bioengineering 11:319. https://doi.org/10.3390/bioengineering11040319
106. Pachetti E, Colantonio S (2024) A systematic review of few-shot learning in medical imaging. Artif Intell Med 156:102949. https://doi.org/10.1016/j.artmed.2024.102949
107. Kazerooni AF, Khalili N, Liu X, et al (2025) BraTS-PEDs: Results of the Multi-Consortium International Pediatric Brain Tumor Segmentation Challenge 2023. Mach Learn Biomed Imaging 3:72–87. https://doi.org/10.59275/j.melba.2025-f6fg

108. info@sagebase.org SB BraTS-PED. https://www.synapse.org/Synapse:syn51514108. Accessed 28 Jan 2026
109. Thummerer A, van der Bijl E, Galapon Jr A, et al (2023) SynthRAD2023 Grand Challenge dataset: Generating synthetic CT for radiotherapy. Med Phys 50:4664–4674. https://doi.org/10.1002/mp.16529
110. SynthRAD2023 Grand Challenge dataset: synthetizing computed tomography for radiotherapy. https://zenodo.org/records/7260705. Accessed 28 Jan 2026
111. Xu Y, Ding Y, Sun H, et al (2025) HyKid: An Open MRI Dataset with Expert-Annotated Multi-Structure and Choroid Plexus in Pediatric Hydrocephalus
112. info@sagebase.org SB HyKid. https://www.synapse.org/Synapse:syn68544889/wiki/633233. Accessed 28 Jan 2026
113. Pham HH, Nguyen NH, Tran TT, et al (2023) PediCXR: An open, large-scale chest radiograph dataset for interpretation of common thoracic diseases in children. Sci Data 10:240. https://doi.org/10.1038/s41597-023-02102-5
114. VinDr-PCXR: An open, large-scale pediatric chest X-ray dataset for interpretation of common thoracic diseases. https://physionet.org/content/vindr-pcxr/1.0.0/. Accessed 28 Jan 2026
115. Jernigan TL, Brown SA, Dale AM, et al (2025) ABCD Study(R) Data Release 6.0
116. Pohl K ABCD Neurocognitive Prediction Challenge 2019. https://nda.nih.gov/edit_collection.html?id=3104. Accessed 28 Jan 2026
117. Di Martino A, Yan C-G, Li Q, et al (2014) The autism brain imaging data exchange: towards a large-scale evaluation of the intrinsic brain architecture in autism. Mol Psychiatry 19:659–667. https://doi.org/10.1038/mp.2013.78
118. ABIDE. https://fcon_1000.projects.nitrc.org/indi/abide/abide_I.html. Accessed 28 Jan 2026
119. ABIDE. https://fcon_1000.projects.nitrc.org/indi/abide/abide_II.html. Accessed 28 Jan 2026
120. Feczko E, Stoyell SM, Moore LA, et al (2025) Baby Open Brains: An open-source dataset of infant brain segmentations. Sci Data 12:1423. https://doi.org/10.1038/s41597-025-05404-y
121. Baby Open Brains (BOBs) Repository. https://bobsrepository.s3.amazonaws.com/index.html. Accessed 28 Jan 2026
122. Reynolds JE, Long X, Paniukov D, et al (2020) Calgary Preschool magnetic resonance imaging (MRI) dataset. Data Brief 29:105224. https://doi.org/10.1016/j.dib.2020.105224
123. Reynolds J, Long X, Paniukov D, et al (2019) Calgary Preschool MRI Dataset. https://osf.io/axz5r. Accessed 28 Jan 2026
124. Alexander LM, Escalera J, Ai L, et al (2017) An open resource for transdiagnostic research in pediatric mental health and learning disorders. Sci Data 4:170181. https://doi.org/10.1038/sdata.2017.181
125. Data-Portal-Project. https://fcon_1000.projects.nitrc.org/indi/cmi_healthy_brain_network/MRI_EEG.html. Accessed 28 Jan 2026
126. Holland S (2015) Cincinnati MR Imaging of Neurodevelopment (C-MIND). https://www.nitrc.org/projects/cmind_2014/. Accessed 28 Jan 2026
127. Holland S (2015) Cincinnati MR Imaging of Neurodevelopment (C-MIND). https://nda.nih.gov/edit_collection.html?id=2329. Accessed 28 Jan 2026
128. Power JD, Barnes KA, Snyder AZ, et al (2012) Spurious but systematic correlations in functional connectivity MRI networks arise from subject motion. NeuroImage 59:2142–2154. https://doi.org/10.1016/j.neuroimage.2011.10.018

129. Dosenbach NUF, Nardos B, Cohen AL, et al Prediction of Individual Brain Maturity Using fMRI. https://fcon_1000.projects.nitrc.org/indi/retro/Power2012.html. Accessed 28 Jan 2026
130. Edwards AD, Rueckert D, Smith SM, et al (2022) The Developing Human Connectome Project Neonatal Data Release. Front Neurosci 16:886772. https://doi.org/10.3389/fnins.2022.886772
131. Consolidated release, now including data from both fetuses and neonates. https://biomedia.github.io/dHCP-release-notes/. Accessed 28 Jan 2026
132. Chambers C, Nelson C, Dale A, et al (2025) HEALthy Brain and Child Development (HBCD) Study. https://hbcdstudy.org/. Accessed 19 Nov 2025
133. Chambers C, Nelson C, Dale A, et al HBCD Study Data Release 1.1. https://www.nbdc-datahub.org/data-tools-lasso. Accessed 28 Jan 2026
134. Akinci D'Antonoli T, Todea R-A, Leu N, et al (2023) Development and Evaluation of Deep Learning Models for Automated Estimation of Myelin Maturation Using Pediatric Brain MRI Scans. Radiol Artif Intell 5:e220292. https://doi.org/10.1148/ryai.220292
135. Akinci D'Antonoli T, Todea R-A, Datta A, et al Large dataset of infancy and early childhood brain MRIs (T1w and T2w). https://zenodo.org/records/8055666. Accessed 28 Jan 2026
136. Wang J, Lytle MN, Weiss Y, et al (2022) A longitudinal neuroimaging dataset on language processing in children ages 5, 7, and 9 years old. Sci Data 9:4. https://doi.org/10.1038/s41597-021-01106-3
137. A longitudinal neuroimaging dataset on language processing in children ages 5, 7, and 9 years old - OpenNeuro. https://openneuro.org/datasets/ds003604/versions/1.0.7. Accessed 28 Jan 2026
138. Abdi AH, Kasaei S, Mehdizadeh M (2015) Automatic segmentation of mandible in panoramic x-ray. J Med Imaging 2:044003. https://doi.org/10.1117/1.JMI.2.4.044003
139. Abdi A, Kasaei S Panoramic Dental X-rays With Segmented Mandibles. https://data.mendeley.com/datasets/hxt48yk462/2. Accessed 28 Jan 2026
140. Jordan P, Adamson PM, Bhattbhatt V, et al (2021) Pediatric Chest/Abdomen/Pelvic CT Exams with Expert Organ Contours (Pediatric-CT-SEG). https://doi.org/10.7937/TCIA.X0H0-1706. Accessed 19 Nov 2025
141. PEDIATRIC-CT-SEG. In: Cancer Imaging Arch. TCIA. https://www.cancerimagingarchive.net/collection/pediatric-ct-seg/. Accessed 28 Jan 2026
142. Popa M, Vișa GA, Șofariu CR (2025) PediMS: A Pediatric Multiple Sclerosis Lesion Segmentation Dataset. Sci Data 12:1184. https://doi.org/10.1038/s41597-025-05346-5
143. PediMS: A Pediatric Multiple Sclerosis Lesion Segmentation Dataset. https://figshare.com/articles/dataset/PediMS_A_Pediatric_Multiple_Sclerosis_Lesion_Segmentation_Dataset/28701065/1. Accessed 28 Jan 2026
144. Halabi SS, Prevedello LM, Kalpathy-Cramer J, et al (2019) The RSNA Pediatric Bone Age Machine Learning Challenge. Radiology 290:498–503. https://doi.org/10.1148/radiol.2018180736
145. RSNA Bone Age. https://www.kaggle.com/datasets/kmader/rsna-bone-age. Accessed 28 Jan 2026
146. Jernigan TL, Brown TT, Hagler DJ, et al (2016) The Pediatric Imaging, Neurocognition, and Genetics (PING) Data Repository. NeuroImage 124:1149–1154. https://doi.org/10.1016/j.neuroimage.2015.04.057
147. Bartsch H Pediatric Imaging, Neurocognition, and Genetics (PING). https://nda.nih.gov/edit_collection.html?id=2607. Accessed 28 Jan 2026

148. CCDI Hub - Childhood Cancer Data Initiative Hub. https://ccdi.cancer.gov/. Accessed 28 Jan 2026
149. Children’s Brain Tumor Network - United We Cure. https://cbtn.org/. Accessed 28 Jan 2026
150. Pediatric Data Collections and Analysis. In: Cancer Imaging Arch. TCIA. https://www.cancerimagingarchive.net/pediatric-data-collections-and-analysis/. Accessed 28 Jan 2026
151. Grand Challenge. In: Gd.-Challengeorg. https://grand-challenge.org/. Accessed 28 Jan 2026
152. IDC. https://portal.imaging.datacommons.cancer.gov/. Accessed 28 Jan 2026
153. Find Open Datasets and Machine Learning Projects | Kaggle. https://www.kaggle.com/datasets. Accessed 28 Jan 2026
154. Zenodo. https://zenodo.org/. Accessed 28 Jan 2026
155. Medical Imaging | IEEE DataPort. https://ieee-dataport.org/topic-tags/medical-imaging. Accessed 28 Jan 2026
156. NIMH Data Archive - NDA. https://nda.nih.gov/. Accessed 28 Jan 2026
157. NITRC: Welcome. https://www.nitrc.org/. Accessed 28 Jan 2026
158. AI Challenges. https://www.rsna.org/artificial-intelligence/ai-image-challenge. Accessed 28 Jan 2026
159. Klontzas ME, Groot Lipman KBW, Akinci D’ Antonoli T, et al (2025) ESR Essentials: common performance metrics in AI—practice recommendations by the European Society of Medical Imaging Informatics. Eur Radiol. https://doi.org/10.1007/s00330-025-11890-w
160. Hui M (Lucy), Sacoransky E, Chung A, Kwan BY (2025) Exploring the integration of artificial intelligence in radiology education: A scoping review. Curr Probl Diagn Radiol 54:332–338. https://doi.org/10.1067/j.cpradiol.2024.10.012
161. Verghese BG, Iyer C, Borse T, et al (2025) Modern artificial intelligence and large language models in graduate medical education: a scoping review of attitudes, applications & practice. BMC Med Educ 25:730. https://doi.org/10.1186/s12909-025-07321-5
162. Kim HE, Cosa-Linan A, Santhanam N, et al (2022) Transfer learning for medical image classification: a literature review. BMC Med Imaging 22:. https://doi.org/10.1186/s12880-022-00793-7
163. Misera L, Müller-Franzes G, Truhn D, Kather JN (2024) Weakly Supervised Deep Learning in Radiology. Radiology 312:e232085. https://doi.org/10.1148/radiol.232085
164. Noh S, Lee B-D (2025) A narrative review of foundation models for medical image segmentation: zero-shot performance evaluation on diverse modalities. Quant Imaging Med Surg 15:5825858–5825858. https://doi.org/10.21037/qims-2024-2826
165. Peretti L, Donatelli G, Cencini M, et al (2023) Generating Synthetic Radiological Images with PySynthMRI: An Open-Source Cross-Platform Tool. Tomography 9:1723–1733. https://doi.org/10.3390/tomography9050137
166. Pfaehler E, Euba D, Rinscheid A, et al (2022) CNNs for automatic image quality control and EARL compliance of PET images. EJNMMI Phys 9:53. https://doi.org/10.1186/s40658-022-00468-w
