# Supplementary material for: Application of artificial intelligence in paediatric oncology imaging
Source: Pediatr Radiol. 2026 Apr 28;56(9):1975–95. doi: 10.1007/s00247-026-06606-1 (PMC13546343; doi:10.1007/s00247-026-06606-1)
Supplement: Supplementary file 2 [file 247_2026_6606_MOESM2_ESM.pdf]

De Donno et al (2026) Application of artificial intelligence in paediatric oncology imaging. Pediatr Radiol. https://doi.org/10.1007/s00247-026-06606-1

Supplementary Material 2.
Literature search methodology

To map the current research landscape, a comprehensive literature search was conducted for this narrative review, pairing core topics with key terms such as "paediatric" or "children", "oncology", "tumor", or "cancer" and “AI”, “DL”, “ML” “deep learning” or “machine learning”. Searches were performed primarily in Google Scholar and PubMed, supplemented by reference mining of relevant articles. The retrieved articles were screened by title and abstract for relevance, with the most pertinent publications selected for in-depth review. Where paediatric-specific literature was limited, the search was expanded to include studies from adult populations or non-oncological fields where methodological or conceptual insights were potentially transferable to paediatric oncology. Recent publications (2020 onwards) were prioritised, but older studies were included where necessary to provide context or methodological grounding. In addition, a list of publicly available datasets and platforms relevant to paediatric oncology imaging was compiled, as summarised in **Table 2** and **Table 3** of the main text. Dataset identification was conducted separately using cited public repositories and targeted web searches. A summary of the primary search terms used for each topic is provided in **Table 3**.
